# Men and women differ in their perception of gender bias in research institutions

**DOI:** 10.1371/journal.pone.0225763

**Published:** 2019-12-05

**Authors:** Judit García-González, Patricia Forcén, Maria Jimenez-Sanchez

**Affiliations:** 1 Wom = n Equity & Research Committee, Society of Spanish Researchers in the United Kingdom (SRUK/CERU), International House, 12 Constance Street, London, United Kingdom; 2 School of Biological and Chemical Sciences, Queen Mary, University of London, London, United Kingdom; 3 Department of Basic and Clinical Neuroscience, Maurice Wohl Clinical Neuroscience Institute, Institute of Psychiatry, Psychology & Neuroscience, King’s College London, London, United Kingdom; Northwestern University, UNITED STATES

## Abstract

There is extensive evidence of gender inequality in research leading to insufficient representation of women in leadership positions. Numbers revealing a gender gap in research are periodically reported by national and international institutions but data on perceptions of gender equality within the research community are scarce. In the present study, a questionnaire based on the British Athena Survey of Science, Engineering and Technology (ASSET 2016) was distributed among researchers working in Spain. Consistent with the original UK-based study, women in research perceived a greater degree of gender inequality than men. This difference was consistent from junior to senior positions, within public and private universities as well as research centres, and across all research disciplines. When responses were compared with the existing UK-based questionnaire, researchers in Spain felt that women and men are treated more equally in the workplace, yet they perceived their home departments to be less supportive regarding matters of gender equality. The results of this study provide clear evidence that men and women do not share the same perceptions of gender equality in science and that their differing perceptions are relatively consistent across two major European countries. The fact that men occupy the majority of senior positions while not perceiving the same inequality as women do, may be critical when it comes to ensuring the fair ascent of women to senior positions in an academic system. These data encourage the implementation of measures to ensure that both men and women are aware of gender biases in research.

## Introduction

Worldwide, women represent 53% of bachelor’s and master’s graduates. Parity drops at the PhD level (43% women vs 57% men) and even more at postgraduate level, where only 28% of research positions are occupied by women [1]. This gender gap is more noticeable at the senior level, with a lower representation of women in leadership positions and consequently in decision- and policy-making. She Figures 2015, a report that investigates gender equality in research and innovation in Europe [2], showed that only 21% of grade A, top-level researchers were women and, strikingly, numbers have not improved much from the 20% observed in 2010. In the Spanish academic system, the representation of women is nearly identical to that of the rest of the EU (40.8% vs 41.0%), and women occupy 21.0% of senior positions in Spain vs 20.9% in the EU [2,3].

Gender perceptions may influence women’s ascent to senior positions [4]. Women are perceived as worse scientific leaders [5,6] and are stereotyped as not possessing the innate talent that is required in some fields [7]. These and other gender stereotypes may explain why women receive similar levels of research funding when they are judged on the quality of their research but less funding when judged on the excellence of the researcher [8], are less frequently invited to conferences [9,10], are less likely to be selected for scientific awards [11,12], are less represented on editorial boards [13], their work is less likely to be cited [14], they have less chances of being invited to participate in peer review [14,15], and they have a more restricted access to influential networks [16]. In 2015, Handley *et al* reported that men do not recognise the presence of gender bias in research to the same extent as women: when men and women were asked to read an abstract from a study reporting gender bias in research, men tended to evaluate this study less favourable, suggesting reluctance of men to acknowledge gender bias. The gender difference was more prominent among academics working in science, technology, engineering and mathematics (STEM) [17]. Moreover, many women’s choices of undergraduate discipline are dependent on the potential discrimination that is anticipated in each field [18]. A lack of understanding of these issues, especially at the senior level, will likely result in fewer measures put in place to tackle them. It is therefore necessary to understand how gender biases are perceived by researchers in their workplace, and, importantly, whether gender, seniority, research area and type of institution influence these perceptions. While reports are published periodically to evaluate the current gender situation in science and its evolution over the years [1–3,19], much less is known about researchers’ perceptions of gender equality.

The Athena Survey of Science, Engineering and Technology (ASSET) 2016 [20] was commissioned by The Royal Society, Royal Academy of Engineering, Royal Society of Biology and The Academy of Medical Sciences and managed by the Equality Challenge Unit (ECU) [21] to assess experiences, expectations and perceptions in science, technology, engineering, mathematics and medicine (STEMM) in academia in the United Kingdom (UK). The survey, which expanded from previous iterations of the survey, had 4,869 respondents and covered six aspects of British academics’ working life: perception of gender equality, recruitment, job and career, caring responsibilities, training and leadership, and promotion and development. On average, men felt that the department where they worked was more committed to gender equality than women did. Also, although differences were relatively small, women perceived that men had an advantage regarding the allocation of tasks and resources related to career development, while men’s perceptions on this topic were more neutral.

In Spain, while public organisations such as the Spanish Research Council (CSIC) and the Women and Science Unit of the Spanish Ministry of Science, Innovation and Universities publish periodic reports of statistics regarding women in research [3,19,22], to the best of our knowledge, there has not been a formal assessment of perceptions on gender equality. Moreover, policies to encourage and recognise commitment to advancing the careers of women have not yet being implemented systematically, in contrast to the UK, where charters such as Athena SWAN (Scientific Women's Academic Network) [23] have been active for more than ten years. The present study seeks to understand gender perceptions and experiences among researchers in Spanish academic institutions, and to compare these with the perceptions of researchers working in their British counterparts. A questionnaire with items adapted from the ASSET 2016 [20] (S1 Table) was distributed among researchers working in both public and private universities and public research institutes in Spain [24]. The effects of respondents’ gender, seniority, type of institution and research area on their perceptions of gender equality were systematically assessed, and the results of this survey were then compared with those of the ASSET 2016. Data from our survey show that men and women differ in the perceptions of gender equality and that findings are consistent across research areas, type of institutions and researchers’ positions. Our findings largely agree with those obtained from respondents in the UK, while highlighting differences in how researchers in Spain perceive less institutional support for gender-related issues.

## Methods

### Participants

A total of 2,619 individuals were contacted via email through their institutions or through the Society of Spanish Researchers in the UK (SRUK/CERU). Of these, we analysed the data provided by 2,255 respondents that were currently working in Spain and discarded the data from individuals that did not reach the end of the survey. To ensure that our sampling method did not introduce a non-response bias in our analyses, we compared responses from those that did not complete the survey with those that completed it and found no differences between them (S2 Table for women and S3 Table for men). 10 individuals younger than 21 were discounted. While this survey included the opportunity for respondents to indicate that they would prefer not to disclose their gender (n = 11), the data presented are limited to those respondents who identified themselves as either men or women. The final sample for analysis contained 1,295 adults from 63 institutions (see S4 Table for a complete list of the institutions represented in the survey), of which 36% (n = 469) were men and 64% (n = 826) were women. For more details of the sample used in the study, see Table 1 and S1 Appendix.

### Research ethics

The data in this study were analyzed anonymously. Data were collected through the website surveymonkey.com. At the beginning of the survey, all participants were informed about the purpose of the questionnaire and the anonymisation of their data. Responses were obtained between 5 February 2018 and 4 May 2018. Participants were given the option of not responding at each question. We only included data from participants older than 21 years old.

### Measures

The present report is part of a wider survey to explore the perceptions and experiences of gender equality of academics working in STEMM, as well as in the arts, humanities, social sciences, business and law (AHSSBL) in Spain. Items included in the original survey were adapted from the Athena Survey of Science, Engineering and Technology (ASSET), managed by the Equality Challenge Unit [20]. The survey was circulated in English to ensure that the questions had the same meaning in both countries. In this study, only the responses relevant to the perception of gender biases were analysed. A description of the survey questions that were adapted from the ASSET survey and analysed in this study, their variable names and scales used is provided in S1 Table.

The measurement of gender equality in research is multidimensional. In this case, two dimensions of gender equality were explored: *perceptions of gender equality in departments* where respondents work and *perceptions of gender equality in the allocation of tasks and resources*. *Perceptions of gender equality in departments* were assessed using six statements such as “My department is committed to promoting gender equality” or “My department is (or would be) responsive to concerns about gender equality”. Each statement was rated using a 7-point scale ranging from 1 = “Strongly disagree” to 7 =“Strongly agree”. *Perceptions of gender equality in the allocation of tasks and resources* were assessed using 15 items, such as “Invitations to conferences”, “Appointments to editorships” or “Allocation of teaching”. Each item was evaluated using a 7-point scale ranging from 1 = “Much easier for a woman” to 7 = “Much easier for a man” (S1 Table).

### Analyses

We performed Principal Component Analysis (PCA) to confirm that the two previously-described dimensions of gender equality are present in the Spanish research system. PCA calculates the correlating variation among a set of observed variables (items) to identify underlying latent variables (dimensions/constructs) by obtaining the covariance matrix of the variables, and then its eigenvectors and the corresponding eigenvalues. Cronbach’s alpha [25] was used to examine the internal validity of the items for each component. To assess whether respondents’ gender had a significant effect on their perceptions of gender equality, independent samples t-tests were performed for each survey question and for the sum of all items within each dimension. Effect sizes were assessed using Cohen's d [26], where 0.2, 0.5, and 0.8 indicated a small, medium and large effect, respectively. To evaluate the effects of research area, position, type of institution, as well as the interaction between those and the respondents’ gender, two-way ANOVA tests were used (three ANOVA tests were run, one for each factor). Mean, standard deviation and sample sizes for male and female respondents in the UK were obtained from ASSET 2016 and t-tests were carried out separately to compare each question and gender group.

To account for multiple testing when exploring group differences between men and women, a Bonferroni correction was applied based on 21 independent t-tests (one for each question for the Spain based questionnaire) and significance was declared at a threshold of 0.002. For the comparison across countries, a Bonferroni correction was applied based on 38 independent t-tests (19 questions available in both countries stratified by male and female respondents). In this case significance was declared at a threshold of 0.001. Analyses were undertaken using Minitab v.17 and v.18 and R version 3.4.3.

## Results

To assess how researchers working in Spain perceive gender equality, a survey adapted from the ASSET 2016 in the UK, was distributed among researchers working in Spanish universities and research centres. A total of 1,295 complete responses were collected from 63 institutions, of which 36% (n = 469) were men and 64% (n = 826) were women. Respondents’ ages ranged between 21 and 66 or over and represented all stages of the research and academic ladder (Table 1). The survey was composed of two categories: *perceptions of gender equality in departments* and *perceptions of gender equality in the allocation of tasks and resources*. We first confirmed the existence of two defined categories among the questions by performing a principal component analysis (PCA) and their internal reliability was assessed by Cronbach’s alpha. With Cronbach’s alpha values ranging from 0.7 to 0.9, we confirmed that the items within each component were closely related. These results are in line with the ASSET 2016 survey structure, ensuring a reliable comparison between both countries (see S2 Appendix in supporting information and S1 Fig for details on the psychometric analyses).

**Table 1:** Sample characteristics and key frequencies.

	Total N = 1,295 (%)		
***Gender***		***Position***	
Women	826 (63.8%)	**Senior Researcher**	
Men	469 (36.2%)	Head of school/division/dep	30 (2.3%)
		Centre director	16 (1.2%)
***Age***		Professor	132 (10.3%)
21 to 25	95 (7.3%)	Emeritus professor	9 (0.8%)
26 to 30	180 (13.9%)	Reader	189 (14.5%)
31 to 35	151 (11.7%)	Senior Lecturer	84 (6.4%)
36 to 40	162 (12.5%)	Group Leader	96 (7.4%)
41 to 45	155 (11.9%)	**Intermediate career researcher**	
46 to 50	191 (14.7%)	Lecturer	123 (9.6%)
51 to 55	174 (13.4%)	Associate lecturer/ Teaching	48 (3.7%)
56 to 60	108 (8.3%)	assistant	
61 to 65	54 (4.2%)	Research Fellow	41 (3.1%)
66 and over	25 (1.9%)	**Early Career Researcher**	
		Postdoctoral Fellow	70 (5.4%)
***Research Area***		Postdoctoral Research	82 (6.3%)
Biological sciences	378 (28.9%)	Associate	
Medical & Health	196 (15.0%)	Research Assistant	45 (3.5%)
Sciences		Research Technician	15 (1.2%)
Business & Finance	29 (2.2%)	**Research Student**	
Chemical Sciences	83 (6.4%)	PhD student	258 (20.0%)
Earth sciences	30 (2.3%)	Master student	3 (0.2%)
Engineering & computing	164 (12.6%)	Undergraduate student	3 (0.02%)
Humanities & Arts	87 (6.7%)	**Other**	51 (3.9%)
Law	28 (2.1%)		
Maths & physical	155 (11.9%)	***Type of working institution***	
Sciences		Public University	691 (53.4%)
Social sciences	145 (11.1%)	Private University	136 (10.5%)
		Research centres	342 (26.4%)
		Others	126 (9.7%)

We then assessed the impact that gender, position, research area and type of institution may have on perceptions of gender equality in the Spanish academic system. T-tests and two-way analysis of variance (ANOVA) tests were used to assess the effect of these factors as well as the interaction between them and the respondents’ gender. In addition, responses were compared with those from the ASSET 2016 to investigate potential differences in perceptions across Spain and the UK.

### Gender differences in perceptions of gender equality in departments

**Fig 1:**
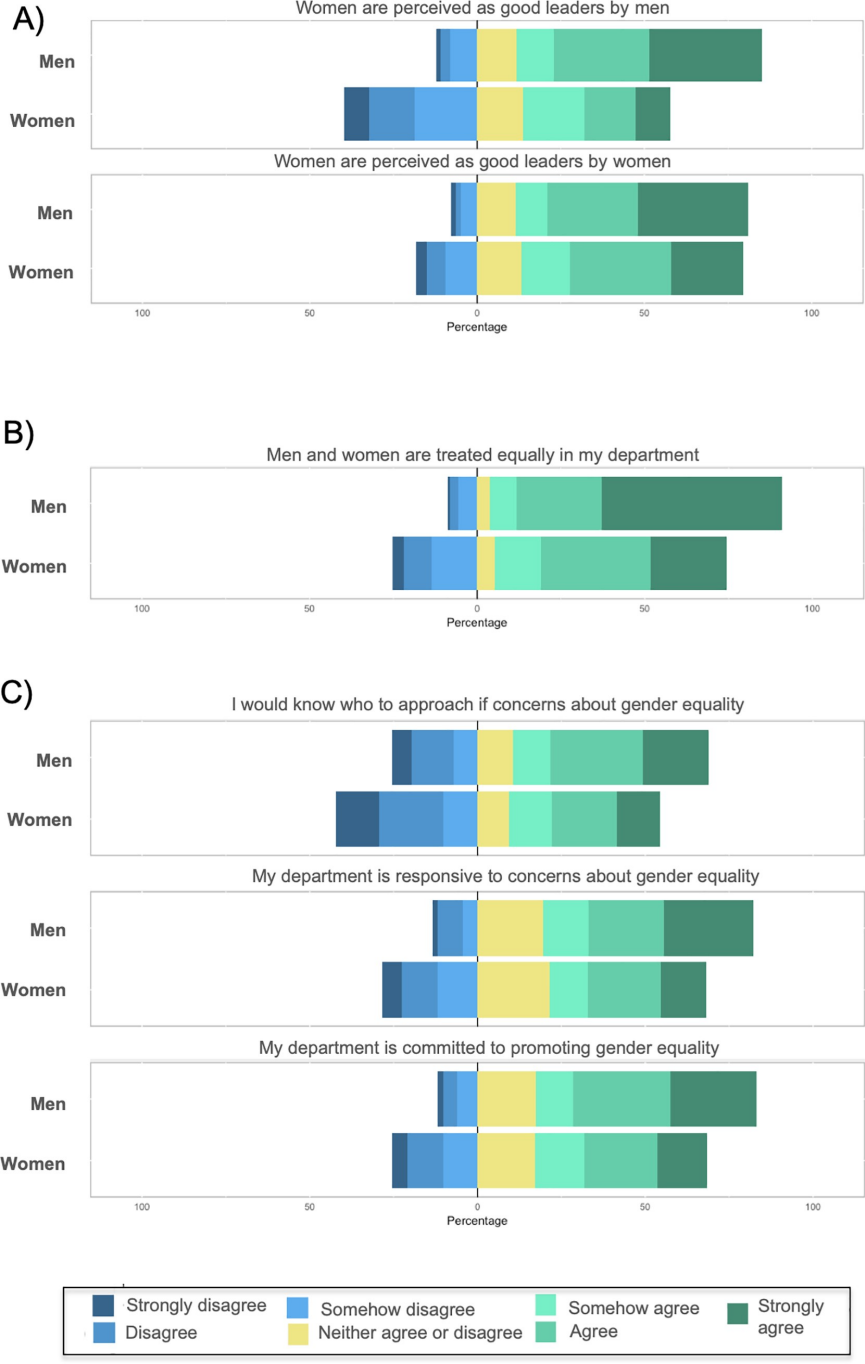
Gender differences in perceptions of gender equality in the respondents’ departments. Graph shows the distribution of responses by gender where responses ranged from 1 = “Strongly disagree” to 7 = “Strongly agree”. The neutral value is 4 = “Neither agree nor disagree”. Sample sizes ranged from 1,287 to 1,293 respondents (n = 465 to 468 men and n = 821 to 826 women). Sample sizes for each question are detailed in S6 Table.

**Fig 2:**
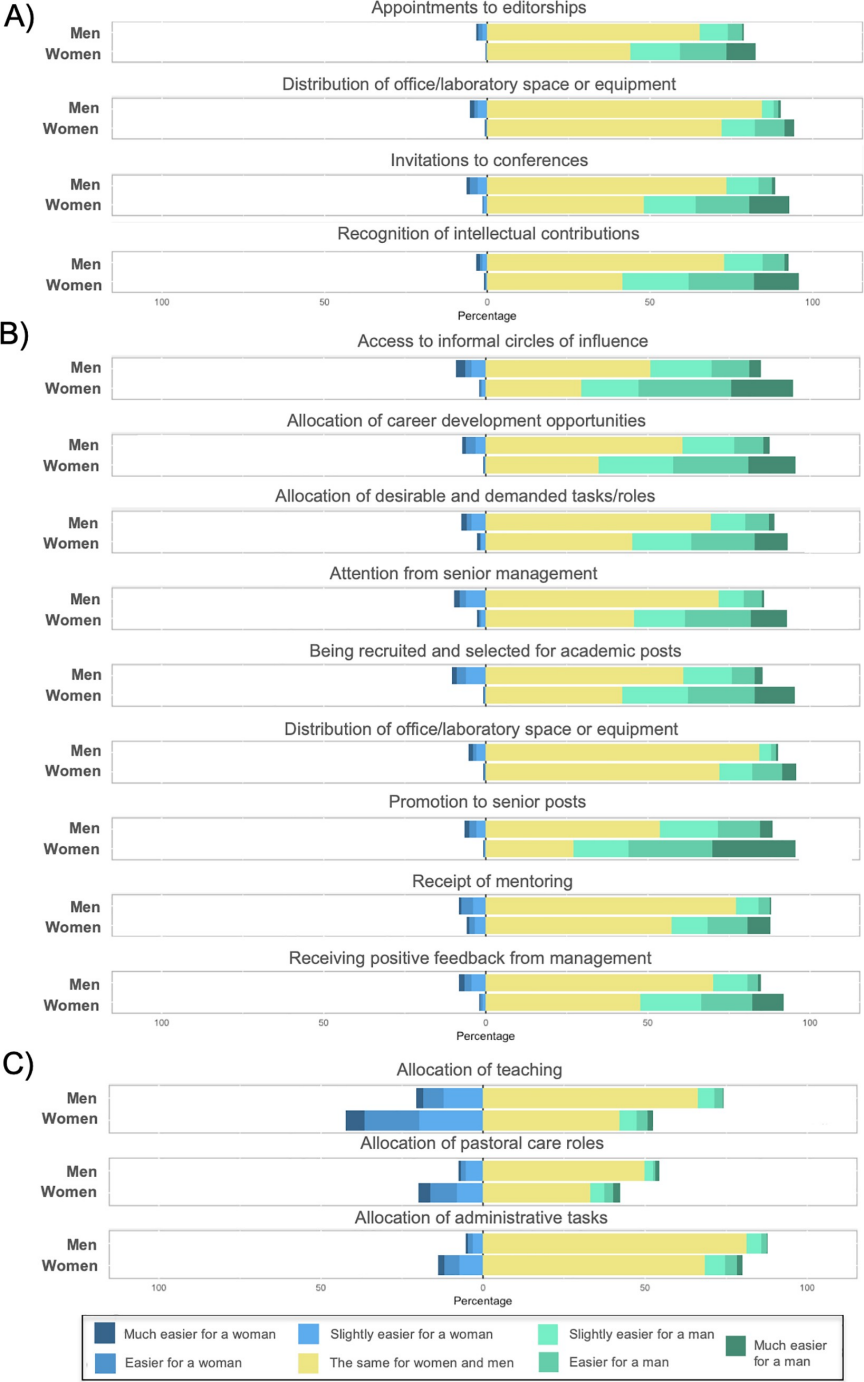
**Gender differences in perceptions of gender equality in the allocation of tasks and resources related to A) markers of esteem, B) professional development and C) additional professional duties.** The item ‘Distribution of office/laboratory space or equipment’ refers to both A) markers of esteem and B) professional development. Graphs show distribution of responses by gender where responses ranged from 1 = “Much easier for a woman” to 7 = “Much easier for a man”. The neutral value is 4 = “Neither agree nor disagree”. See S4 Table for descriptive statistics and t-test results. Sample size ranged from n = 1,259 to 1,287 respondents (n = 455 to 467 men and n = 804 to 821 women). Sample sizes for each question are detailed in S7 Table.

**Fig 3:**
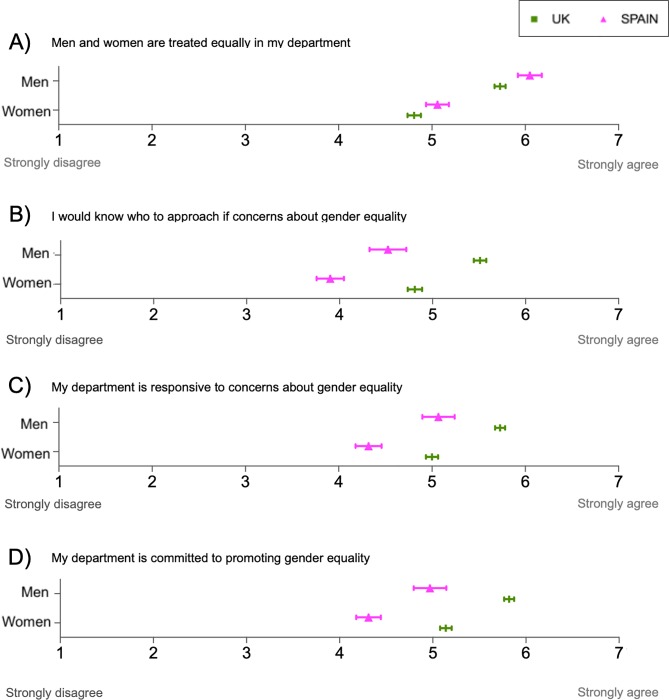
Perceptions of gender equality in the respondents’ departments in the Spanish and British academic systems. Responses range from 1 = “Strongly disagree” to 7 = “Strongly agree”. The neutral value is 4 = “Neither agree nor disagree”. Spanish sample size ranged from 1,297 to 1,303 respondents (n = 467 to 468 men and n = 817 to 826 women). British sample size ranged from 4,804 to 4,862 respondents (n = from 2,466 to 2,491 men and n = from 2,338 to 2,372 women). Sample sizes for each question, country and gender are detailed in S17 Table.

In the first part of the survey, a total of six items were used to evaluate how participants perceived gender equality in their departments in terms of (1) leadership (assessing how well women and men perceive women as leaders (Fig 1A)), (2) equality treatment (assessing whether men and women are treated equally in their departments (Fig 1B)), and (3) promotion of gender equality (investigating whether participants perceived that their departments have measures in place to promote gender equality (Fig 1C)). Perceptions of gender equality in the respondents’ department was overall lower for women, with average score across the six items close to neutral (*M* = 4.44, *SD* = 1.93) compared to men, who perceived their departments are somewhat committed to gender equality (*M* = 5.18, *SD* = 2.13) (*p*<0.002, S6 Table). The distribution of responses for this category also showed that, despite the high variability in responses within each gender, most of men responses were 6 = ‘Agree’ and 7 = ‘Strongly agree’ (that there is gender equality in their departments), whereas women responses were more variable and a larger percentage of them failed to perceive gender equality (1 = ‘Strongly disagree’, 2 = ‘Disagree’ and 3 = ‘Somehow disagree’) (Fig 1).

The largest gender differences were observed when participants were asked about leadership perception (Fig 1A). Although both women and men mostly agreed with the statement ‘Women are perceived as good leaders by women’, there was a slight shift in the distribution of responses towards a more negative perception by women (*M* = 5.05, *SD* = 2.26) than men (*M* = 5.40, *SD* = 1.76). The difference between women and men’s perception was more striking for the question “Women are perceived as good leaders *by women*”, which showed that women felt that women’s leadership abilities are less recognised by men (*M* = 4.03, *SD* = 1.88) (*p*<0.002, S6 Table).

When respondents were asked whether men and women receive equal treatment in their departments (Fig 1B), 87% of men agreed (strongly agree/agree/somehow agree). In contrast, women’s perceptions of equality were significantly lower and only a 69% agreed with that statement, while 25% of them strongly disagreed, disagreed or somehow disagreed with the equality of the treatment received. With an average of 6.05 (*SD* = 1.41) for men versus 5.06 (*SD* = 1.79) (*p*<0.002, S6 Table*)* for women, female researchers perceived less gender equality in the treatment provided by their departments.

To evaluate whether participants perceived that their departments have measures in place to promote gender equality, we used three items that included questions such as “I would know who to approach if I had concerns about gender equality” or “My department is responsive to concerns about gender equality” (Fig 1C). For both men and women, item means ranged between 3.90 (*SD = 2*.*15*) and 5.07 (*SD = 1*.*90*) (scores of 3, 4 and 5 correspond to “Somehow disagree”, “Neither agree nor disagree” and “Somehow agree”, respectively). For these three items, women perceived that their departments had significantly lower commitment to promote gender equality compared to men (*p*<0.002, S6 Table).

Overall, these results show that in the Spanish research system men have a more positive perception about their departments treatment and commitment to gender equality than women do. Importantly, we found that women felt they are not valued as good leaders by men.

### Gender differences in perceptions of gender equality in the allocation of tasks and resources

To evaluate whether men and women perceive that the tasks and resources are equally allocated in their departments, 15 tasks and resources were assessed and stratified by: (1) allocation of markers of esteem (Fig 2A), (2) allocation of professional development resources (Fig 2B) and allocation of academic duties (3) (Fig 2C) (S7 Table).

Compared to men, a larger percentage of women perceived that the recognition of intellectual contributions, invitations to conferences, distribution of office/laboratory space or equipment and appointments to editorships, all markers of esteem, are more easily allocated to men (Fig 2A), with mean scores between 4.01 (*SD* = 2.07) and 4.88 (*SD =* 1.43) (S7 Table). However, male respondents mostly rated the allocation of these resources as ‘the same for men and women’, with mean scores between 3.81 (*SD = 1*.*02)* and 4.07 (*SD = 1*.*13*), Fig 2A and S7 Table).

Similarly, most of the men perceived that the allocation of resources related to professional development (Fig 2B and S7 Table) are allocated to men and women with similar ease (*M* = 3.98, *SD* = 1.25). However, a larger proportion of female respondents felt that most of these resources are more easily allocated to men (*M* = 4.75, *SD* = 1.46). Although these differences were subtle, they were statistically significant, with p<0.002 for all of the items individually and when considered together (S7 Table). The most noticeable differences were found when asked about promotion to senior posts or access to circles of influence (women: *M* = 5.29, *SD* = 1.57; men: *M* = 4.24, *SD* = 1.43; p<0.002, S7 Table). Across all the items, the response distribution is markedly shifted between women and men. The percentage of women that think that it is slightly easier, easier or much easier for a man to get these resources ranged between 24 and 65%, in contrast to a smaller fraction of men with similar opinion, between 6 and 34%. For the different questions, between 50 and 84% of men perceived that professional development resources are distributed equally (Fig 2).

The results above contrast with the findings in relation to the allocation of academic duties (Fig 2C). Both women and men perceived that pastoral care roles, or the support provided for the well-being of students and trainees, are allocated more easily to women and no significant differences between genders were observed for this category (S7 Table). They also agreed that the allocation of teaching is more equally distributed (Fig 2C and S7 Table). While there is a general perception that administrative tasks are more easily allocated to women, women perceived this more strongly (women: *M* = 3.25, *SD* = 1.42; men: *M* = 3.60; *SD* = 1.19. *p*<0.002, S7 Table).

Altogether, gender differences were observed for the allocation of all the items referring to professional development and markers of esteem, where women perceived that these are more easily allocated to men while men did not perceive a biased distribution to the same extent. On the contrary, men and women perceived similarly that academic duties (teaching, pastoral care roles and administrative tasks), which are tasks not directly related to research performance, are distributed more easily to women.

### Interaction of gender and research area in perceptions of gender equality

We next determined whether these gender differences may vary across research areas. Results from a two-way ANOVA for gender and research area suggested that overall women and men differences in gender perception were independent of the research discipline, as no gender-by-research area interaction was statistically significant (S8–S10 Tables). When we compared how researchers from different disciplines perceive gender equality in their workplace, we observed a significant main effect of research area only on the items “In general, men and women are treated equally in my department” and “Allocation of pastoral care roles”. Compared to other research areas, women working on law and earth sciences perceived the lowest gender equality regarding the treatment that men and women receive in their departments (S2 Fig). Researchers in the areas of maths and physical sciences are the ones perceiving that pastoral care roles are more easily allocated to women, with mean scores for both women and men of around 2 (i.e. “Easier for a woman”), while law had the most neutral perception, with mean scores above 3 (i.e. “Slightly easier for a woman”) (S3 Fig). It is worth noting that law and earth sciences are the research areas with the lowest responses and larger samples are needed to reach further conclusions.

### Interaction of gender and position in perceptions of gender equality

To investigate the effect of seniority on perceptions of gender equality, we created four groups of positions according to their experience level (Table 1): senior researcher, intermediate career researcher, early career researcher and research student. Gender and position were included as factors in a two-way ANOVA. Women’s estimates of gender equality were lower than those of men regardless of seniority, as the interaction between position and gender did not reach statistical significance for any item (S11–13 Tables). Similar results were obtained when the interaction was done between age and gender (S11–13 Tables). Only for the item “receiving positive feedback from management” the effect of gender differed by age (S13 Table)

The only significant main effect of position was found on the items “If I had concerns about gender equality in my department, I would know who to approach” (S4 Fig), and “Appointment to editorships” and “Allocation of administrative tasks” (S5 Fig). For all three items, junior researchers perceived more gender biases in the allocation of these resources than researchers in more advanced positions.

### Interaction of gender and type of centre in perceptions of gender equality

We observed that perceptions of gender equality in departments and in the allocation of tasks and resources were consistent across research centres, private and public universities. There were no significant main effects of type of centre, nor any interactions between gender and type of centre (S14–S16 Tables), suggesting that the previously-observed gender differences did not vary as a function of the institution where the respondents work.

### Perceptions of gender equality in the Spanish and British academic systems

Overall, results from our survey and from the ASSET 2016 indicate that lower gender equality was perceived by women researchers working at both Spain and the UK. When all the items from the category perceptions of gender equality in the allocation of tasks and resources were considered together, we found no significant differences between countries (S17 Table). In contrast, when the six items for the category perceptions of gender equality in the department were jointly assessed, male and female researchers in the UK perceived greater gender equality than their counterparts in Spain. In both countries, men perceived higher equality in their departments than women, but country differences were consistent across genders with *p*<0.001 (S17 Table).

We then evaluated all the items individually and compared the responses from both surveys. Significant differences in perceptions between participants from Spain and the UK were observed for both genders (*p*<0.001) in 13 items as per t-test (S17 Table). The largest differences were observed for items related to the support provided by the department and the allocation of teaching and pastoral tasks.

Relative to British respondents, researchers from Spanish institutions perceived greater equality in the treatment that men and women receive in their departments (*p*<0.001, S17 Table) (Fig 3A). Conversely, respondents from Spain perceived a lower level of support from their departments concerning issues of gender equality relative to their British counterparts, with *p*<0.001 for the three items (Fig 3B–3D and S17 Table).

**Fig 4:**
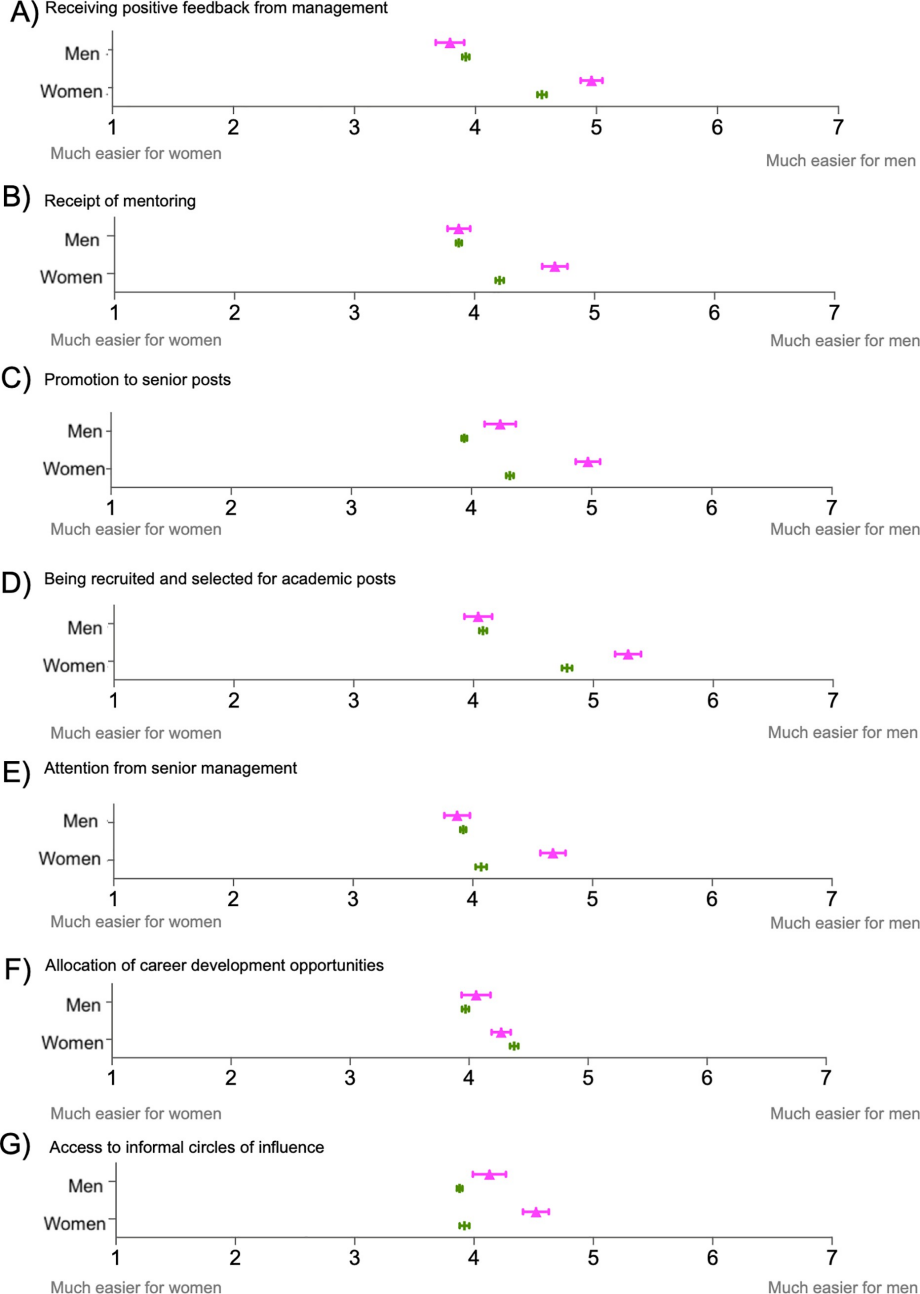
Perceptions of gender equality in the allocation of tasks and resources in the Spanish and British academic systems: professional development. Responses range from 1 = “Much easier for women” to 7 = “Much easier for men”. Spanish sample size ranged from n = 1,279 to 1,287 (n = 46 to 470 men and n = 810 to 827 women). British sample size ranged from 4,814 to 4,824 respondents (n = from 2,467 to 2,477 men and n = from 2,342 to 2,349 women). Sample sizes for each question, country and gender are detailed in S17 Table.

For perceptions of gender equality in the allocation of tasks and resources related to professional development, we observed that differences between Spain and the UK were driven almost exclusively by female respondents (Fig 4). Women working as researchers in Spain perceived to a greater extent that it is easier for a man to be allocated tasks and resources related to professional development such as receiving positive feedback, receipt of mentoring for career decisions, promotion to senior posts, recruitment for academic posts, attention from senior management or access to informal circles of influence (Fig 4). For all these items, significant differences between Spain and the UK were observed for female respondents, where the UK-based respondents perceived higher levels of equality compared to their Spanish counterparts (*p*<0.001).

**Fig 5:**
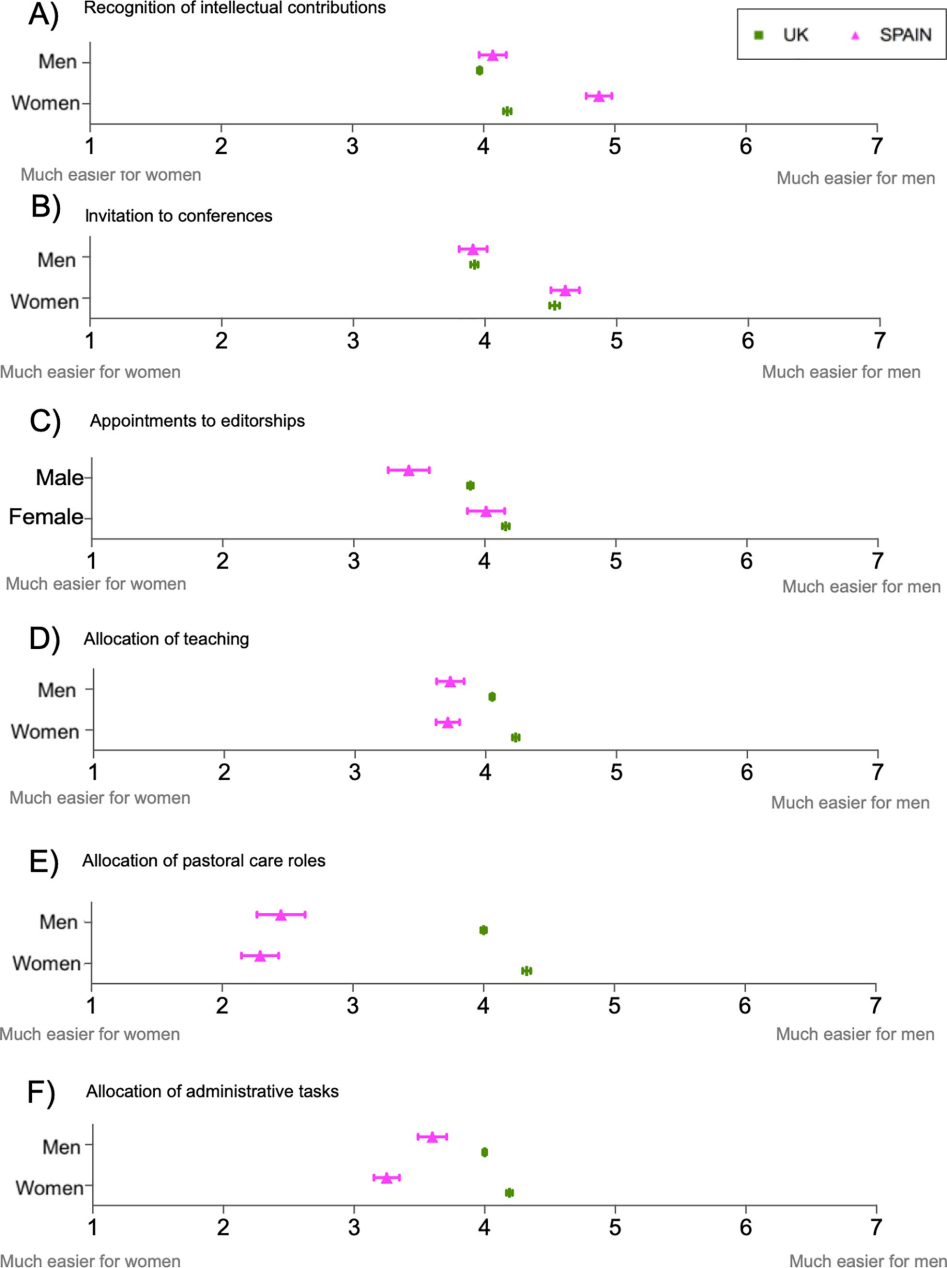
**Perceptions of gender equality in the allocation of tasks and resources in the Spanish and British academic systems: markers of esteem (A-C) and additional professional duties (D-F).** Responses range from 1 = “Much easier for women” to 7 = “Much easier for men”. Spanish sample size ranged from n = 1,259 to 1,286 respondents (n = 455 to 466 men and n = 804 to 820 women). British sample size ranged from 4,722 to 4,813 respondents (n = from 2,433 to 2,476 men and n = from 2,289 to 2,346 women). Sample sizes for each question, country and gender are detailed in S17 Table.

Women in Spain perceived greater inequality in the recognition of intellectual contributions than women in the UK did (p<0.001) (Fig 5A), while no significant differences were observed across countries for other markers of esteem such as invitation to conferences (Fig 5B). Conversely, male Spanish researchers perceived that editorships were more easily allocated to women than British researchers did (p<0.001) (Fig 5C) (S17 Table). Regarding the allocation of teaching, administrative tasks and pastoral roles, Spain-based researchers perceived that these roles are more easily allocated to women while in the UK these would be equally allocated to women and men (*p*<0.001) (Fig 5D–5F and S17 Table). Interestingly, opposite directions in the gender effect were observed between countries for the allocation of administrative tasks and pastoral care roles (Fig 5E–5F).

Despite reaching similar conclusions, both studies also highlight significant differences in gender perception among Spain and the UK. Some of these disparities may arise from inherent characteristics existing between research systems, however it may also underline areas where more work is required to promote gender equality.

## Discussion

The present study is the first one assessing perception of gender equality and comparing it across two major European countries. It provides clear and significant evidence that men and women have a different understanding of the gender gap in academia regardless of the country, research area, junior or senior position and type of academic institution. Our results show that women perceive greater gender inequality than men do and encourage the implementation of measures to increase awareness and address the problem.

Firstly, we evaluated perceptions of gender equality in a sample of 1,295 researchers working in academic positions in Spain. Estimates of gender equality were lower amongst women than men, with small to medium effect sizes, and the largest effect sizes being observed for items related to leadership. Previous research has revealed a systematic, unconscious gender bias that hinders women’s ascent to senior positions [8–16,27,28]. Despite the considerable body of objective scientific evidence, data from our survey shows that male researchers perceive equal gender treatment in their departments, equal access to the resources that are necessary for professional development or that can be viewed as markers of esteem and a stronger commitment from their departments to ensure gender equality. Data from our survey suggests that gender inequalities previously reported in the Spanish research system [3,19,22] are perceived by women researchers in their daily life in their departments but not by men to the same extent. To ensure a fair ascent of women in the academic ladder and fair allocation of resources, it seems necessary that those who occupy senior positions, who are mostly men, have a fair perception of gender inequality.

No significant interactions were observed between academic position or age and gender in our analyses, indicating that men and women of varying ages and seniority shared similar feelings regarding gender equality. Gender inequality has often been explained by a generational effect [29,30], and such an effect was widely cited by respondents when given the option to add comments in our survey (data not shown). These opinions are consistent with reports claiming that women in academia no longer face systematic discrimination [29,30]. However, contrary to this view, EU reports show only a modest increase in the number of women reaching senior positions in recent years [2], while in Spain, the proportion of women occupying senior positions did not change between 2012 and 2017 [3,19]. Results from this survey show that a generational change in perception, which is necessary to reach equality, is not happening in the new generations. Therefore, our data do not support a scenario where perception of gender bias will change over time without a need for intervention.

Our results agree to a large extent with those obtained in the ASSET 2016. Male researchers in both the UK and Spain perceived greater gender equality in their departments compared to female researchers. Interestingly, our analyses also highlighted some key differences in perceptions between the two countries, especially in perceptions related to gender equality in the workplace. While researchers in Spain felt that women and men are treated more equally in the workplace than researchers in the UK did, British departments were perceived as more committed, concerned and responsive to matters of gender equality. Overall perception on the allocation of tasks and resources was more similar between countries, but female respondents based in Spain perceived greater inequality regarding the allocation of resources related to professional development than the UK-based female respondents, while male respondents from both countries perceived no gender inequality. In the UK, the representation of women in the academic system (44.0%) is slightly higher than in Spain (41.0%) and in the EU average (40.8%) [2]. On the contrary, for the representation of women in senior positions, Spain does better, with 21.0% compared to only 17.5% in the UK, which is far from the EU average, 20.9% [2]. We could hypothesize that higher representation of women in senior positions results in greater perceptions of equality among researchers working in Spain. This contrasts with a more positive perception in terms of commitment and support at the workplace in the UK and the resources allocated to professional development.

The UK has been a pioneer in the implementation of awards to encourage and recognise commitment of the institutions to advance the careers of women, such as the Athena SWAN Awards, established by the Equality Challenge Unit (ECU) in 2005. The differences that researchers in Spain and in the UK perceive in terms of institutional support and allocation of resources could be explained by the existence of these measures. Recent evaluations of this program have acknowledged that its implementation has resulted in structural and cultural changes as well as in an effort to advance gender equality in research institutions in the UK [31–33].

The observation of large country differences in the allocation of pastoral care roles and administrative tasks is of special interest. The allocation of these duties has been associated with high workload and low reward [34]. Therefore, some of these differences may arise from the inherent characteristics of both research systems, where the recognition of pastoral roles may not be equally valued. Initiatives such as Athena Swan in the UK, that recognize and value these roles, have potentially led to a more equal distribution in this country.

In the last few years, multiple countries have adopted policies to increment the participation of women in science and to foster their career progression. The Horizon 2020 programme in Europe has incorporated gender in its research and innovation strategy by promoting gender balance in research teams and in decision-making panels and advisory groups, as well as providing funds for initiatives that support gender balance [35]. In the US, the National Science Foundation has invested over $270M to help higher education and STEM-related organizations to support ADVANCE (Organizational Change for Gender Equity in STEM Academic Professions) projects that aim to increase the representation of women in science [36]. In the UK, the Athena SWAN Charter recognises the commitment of academic organisations to gender equality [23], in particular where active policies and specific programmes have been adopted. Gender bias influence decision-making [4,37], therefore how gender biases are perceived by those designing, implementing and assessing these and future measures is a critical aspect to take into consideration [38,39]. At the individual level, perceptions are likely to be shaped during childhood, and working with children to eliminate stereotypes may help eliminating women and men differences in perception from early on [40]. Studies in the social psychology field have shown that alerting about the existence of a certain bias, may reduce that bias [41–43]. Therefore, increasing self-awareness in adulthood through gender bias and unconscious bias workshops could also help shaping perceptions [44]. It is important to note, that identifying the source of bias is critical for an effective intervention [42] and that effective changes require more than a one-off diversity training [45]. More importantly, institutions need to put in place evidence-based, data-driven measures to ensure that perceptions do not have a negative impact in women’s careers progression [46]. Only by applying policy changes and action plans at multiple levels, we will be able to address and remove institutional, organisational, structural and systemic barriers to full gender equality in research.

The ASSET 2016 provided a valuable resource to evaluate perceptions of gender equality in British STEMM. The current survey represents a further attempt to robustly evaluate such perceptions in a representative sample from a different country, although it was limited by an unequal gender distribution, whereby there were twice as many female as male respondents. In addition, the survey was limited to researchers working in universities (public and private universities) and public research centres. Future efforts to better define policies that benefit the largest number of people should include initiatives that encourage the participation and support of men in gender equality surveys, as well as extending surveys to researchers in the private sector.

The present study represents the first formal comparison of men and women perceptions of gender equality between two European countries. Our data on the researchers based in Spanish institutions largely agree with the observations of the British ASSET 2016, while highlighting important differences in gender perceptions between the two research systems. This and future international surveys should aid the design and implementation of effective measures to drive a cultural change and to close the gender gap in research, by increasing our understanding of gender perceptions in academic environments.

## Supporting information

S1 Appendix Responses “Perceptions in Gender Equality”. (XLSX)

S2 Appendix Psychometric properties of the survey. (PDF)

S1 Fig A) Loading plot of survey where first component is represented vs second component. B) Scree plot of the 21 items included in this analysis. As the number of components increases, the variance (within-group sum of squares) decreases. The elbow at two/three clusters represents the most parsimonious balance between minimum number of clusters that explain most of the variance.
(PDF)

S2 Fig Research area by gender interaction in the perception of gender equality in departments. Item represented in the figure corresponds to “In general, men and women are treated equally in my department”. Graph shows means by gender ranging from 1 =“Strongly disagree” to 7 = “Strongly agree”. Sample size N = 1,293 (N = 468 men and N = 825 women).
(PDF)

S3 Fig Research area by gender interaction in the perception of gender equality in the allocation of pastoral care roles. Figure represents the responses to perceptions of gender equality in the “allocation of pastoral care roles” and shows means by gender ranging from 1 =“Much easier for women” to 7 = “Much easier for men”. Sample size N = 1,259 (N = 455 men and N = 804 women).
(PDF)

S4 Fig Position by gender interaction in the perception of gender equality in departments. Item represented in the figure corresponds to “If I had concerns about gender equality in my department, I would know who to approach” and shows means by gender ranging from 1 =“Strongly disagree” to 7 = “Strongly agree”. Sample size N = 1,291 (N = 468 men and N = 823 women).
(PDF)

S5 Fig Position by gender interaction in the perception of gender equality in the allocation of tasks and resources. A) Appointments to editorships and B) Allocation of administrative tasks. Graph shows means by gender ranging from 1 =“Much easier for a woman” to 7 = “Much easier for a man”. Sample size N = 1,275 to 1,279 (N = from 462 to 463 men and N = from 813 to 816 women).
(PDF)

S1 Table Description of the questions in the survey and variable names. (PDF)

S2 Table Comparison between responses from female participants that did not complete the survey (excluded respondents) and participants included in the analysis (respondents that completed the survey). (PDF)

S3 Table Comparison between responses from male participants that did not complete the survey (excluded respondents) and participants included in the analysis (respondents that completed the survey). (PDF)

S4 Table List of the institutions represented in the sample analysed. (PDF)

S5 Table Cronbach alpha coefficients for each item and whole category. (PDF)

S6 Table Descriptive and t-tests results for perceptions of gender equality in departments. “sd” = standard deviation. “N” = sample size. “df” = degrees of freedom. “95CI” = 95% Confidence intervals.
(PDF)

S7 Table Descriptive and t-tests results for perceptions of gender equality in the allocation of tasks and resources. “sd” = standard deviation. “N” = sample size. “df” = degrees of freedom. “95CI” = 95% Confidence intervals.
(PDF)

S8 Table Research area variable names, sample size for each research area and gender distribution by research area. (PDF)

S9 Table Interaction analysis of gender by research area in perceptions of gender equality in departments. “Df” = degrees of freedom. “Sum Sq” = Total sum of squares. “Mean Sq” = Mean Squares.
(PDF)

S10 Table Interaction analysis of gender by research area in the perceptions of gender equality in the allocation of tasks and resources. “Df” = degrees of freedom. “Sum Sq” = Total sum of squares. “Mean Sq” = Mean Squares.
(PDF)

S11 Table Position variable names, sample size for each position and gender distribution by position. (PDF)

S12 Table Interaction analysis of gender by position in the perceptions of gender equality in departments. “Df” = degrees of freedom. “Sum Sq” = total sum of squares. “Mean Sq” = Mean Squares.
(PDF)

S13 Table Interaction of gender by position in the perceptions of gender equality in allocation of tasks and resources. “Df” = degrees of freedom. “Sum Sq” = total sum of squares. “Mean Sq” = mean Squares.
(PDF)

S14 Table Type of institution variable names, sample size for each type of institution and gender distribution. (PDF)

S15 Table Interaction analysis of gender by type of institution in the perceptions of gender equality in departments. “Df” = degrees of freedom. “Sum Sq” = total sum of squares. “Mean Sq” = mean Squares.
(PDF)

S16 Table Interaction analysis of gender by type of institution in the perceptions of gender equality in allocation of tasks and resources. “Df” = degrees of freedom. “Sum Sq” = Total sum of squares. “Mean Sq” = Mean Squares.
(PDF)

S17 Table Comparison of responses between Spain vs the United Kingdom-based researchers. “N” = Sample size, “M” = mean, “SD” = Standard deviation, “df” = degrees of freedom. Note: The questions “women are perceived as good leaders by women/men” from the Spain based questionnaire are not reported in this analysis, as no equivalent questions were available in ASSET 2016. Significance declared at Bonferroni corrected threshold p = 0.001.
(PDF)
