# Supplementary material for: Men and women differ in their perception of gender bias in research institutions
Source: PLoS One. 2019 Dec 5;14(12):e0225763. doi: 10.1371/journal.pone.0225763 (PMC6894819; doi:10.1371/journal.pone.0225763)
Supplement: S2 Appendix — (PDF) [file pone.0225763.s002.pdf]

Appendix1.

Psychometric properties of the survey

Principal component analysis (PCA)

To better understand the survey structure, we performed a PCA to explore the relationship between the observed variables (items) and the latent variables (dimensions/constructs). Two dimensions/constructs were observed, the first one grouped the items related to *perceptions in gender equality between departments* (explaining 30.7 % of the variance) and the second construct was composed by items related to *perceptions of gender equality in the allocation of tasks and resources* (explaining 12.8% of the variance). The third component explained 6% of the variance, thus a two model solution was confirmed. For the PCA, 28 cases containing missing values in one or more of the items were excluded from the analysis (Fig. S1). We hence concluded that the items provided in this survey are a valid and reliable scale to measure Spanish researchers attitudes towards gender equality.

Cronbach alpha coefficients

We calculated Cronbach alpha values to assess how closely related the items from each factor are as a group. Cronbach alpha values ranging from 0.7 to 0.9 suggest good internal consistency and scale reliability of the survey(1). For *perceptions about gender equality in the allocation of tasks and resources* alpha values ranged from 0.85 to 0.88. For *perceptions about gender equality in departments*, alpha values ranged from 0.72 to 0.77. These results suggest that the questions for both dimensions are

measuring their corresponding latent variables. Detailed Cronbach alpha coefficients can be found in Table S2.

References

1. Santos JRA. Cronbach’s Alpha: A Tool for Assessing the Reliability of Scales. Journal of Extension. 1999 Apr; 37(2).
