# Supplementary figures and images for: Men and women differ in their perception of gender bias in research institutions
Source: PLoS One. 2019 Dec 5;14(12):e0225763. doi: 10.1371/journal.pone.0225763 (PMC6894819; doi:10.1371/journal.pone.0225763)

A)

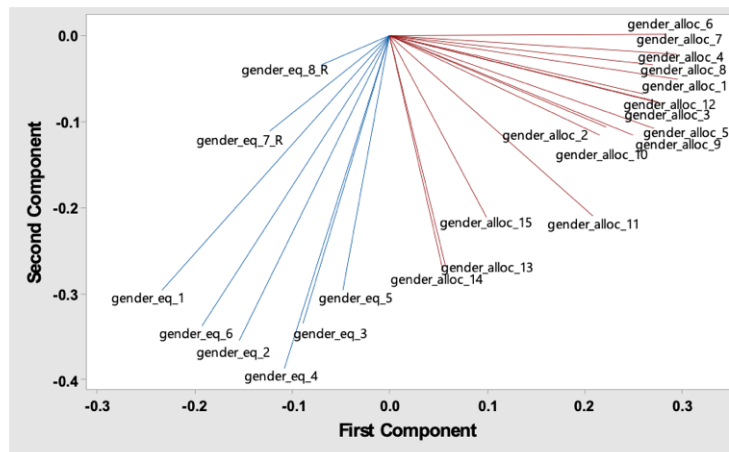

B)

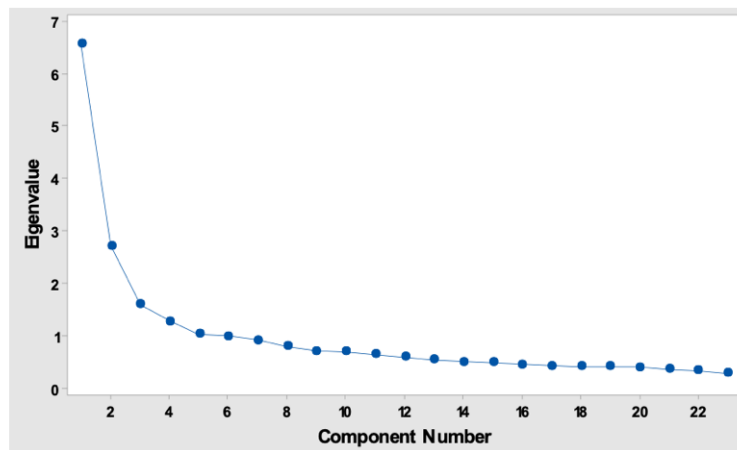

Supplement: S1 Fig — A) Loading plot of survey where first component is represented vs second component. B) Scree plot of the 21 items included in this analysis. As the number of components increases, the variance (within-group sum of squares) decreases. The elbow at two/three clusters represents the most parsimonious balance between minimum number of clusters that explain most of the variance. (PDF) [file pone.0225763.s003.pdf]

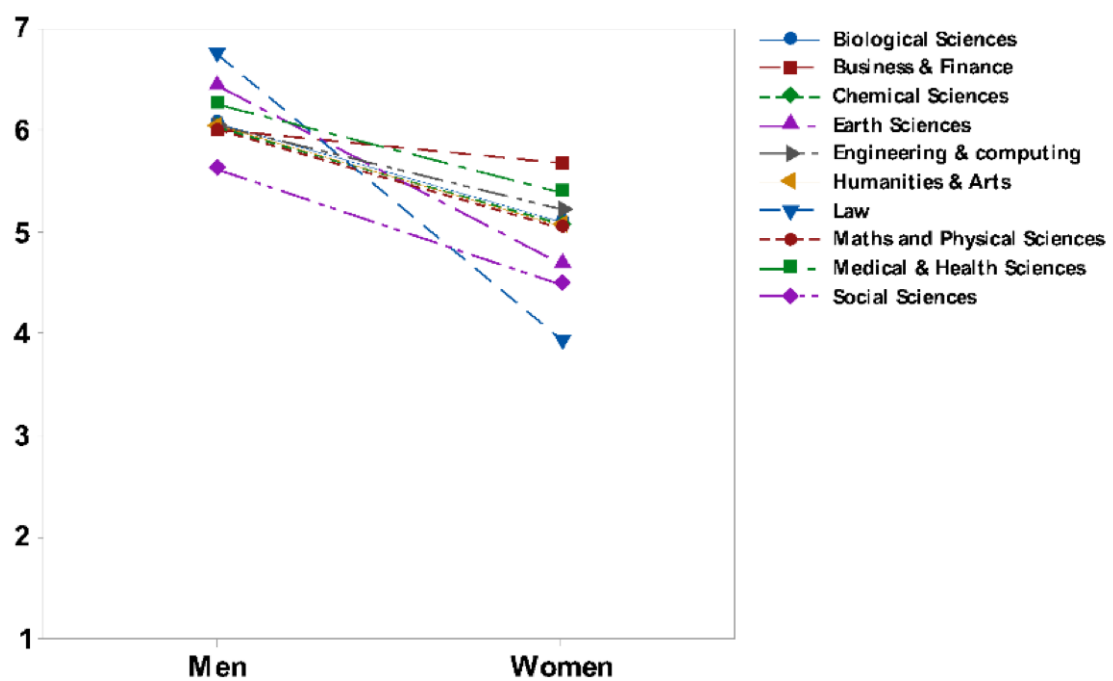

Supplement: S2 Fig — Item represented in the figure corresponds to “In general, men and women are treated equally in my department”. Graph shows means by gender ranging from 1 =“Strongly disagree” to 7 = “Strongly agree”. Sample size N = 1,293 (N = 468 men and N = 825 women). (PDF) [file pone.0225763.s004.pdf]

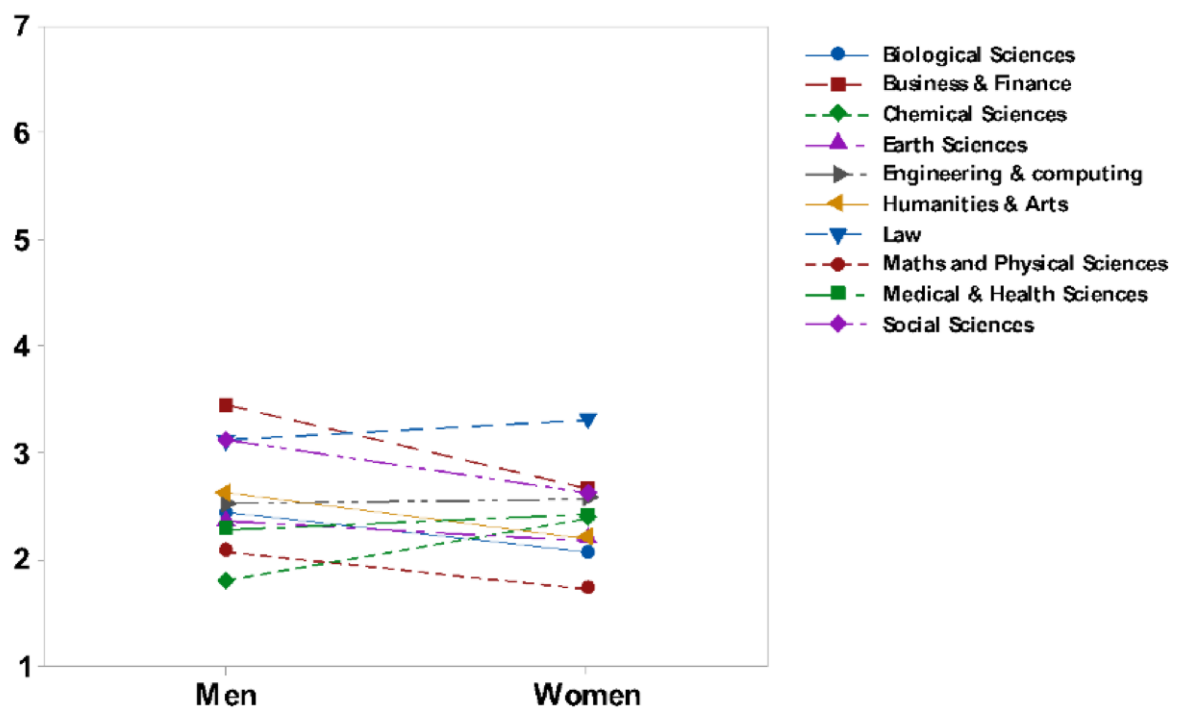

Supplement: S3 Fig — Figure represents the responses to perceptions of gender equality in the “allocation of pastoral care roles” and shows means by gender ranging from 1 =“Much easier for women” to 7 = “Much easier for men”. Sample size N = 1,259 (N = 455 men and N = 804 women). (PDF) [file pone.0225763.s005.pdf]

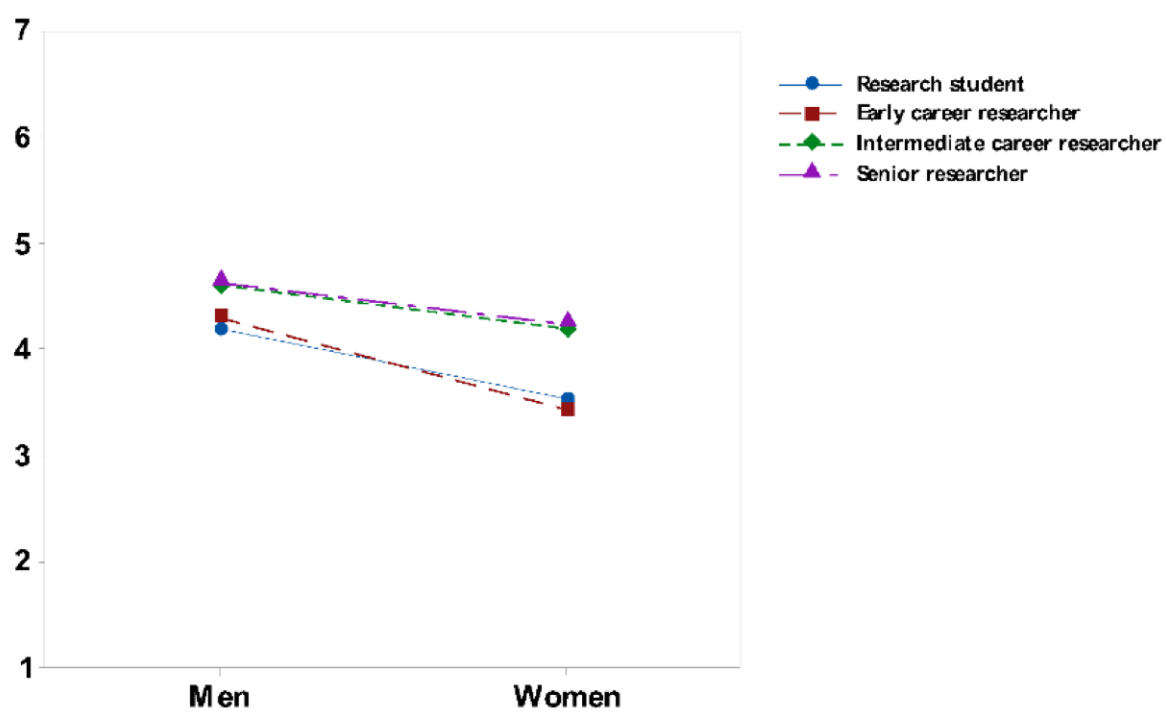

Supplement: S4 Fig — Item represented in the figure corresponds to “If I had concerns about gender equality in my department, I would know who to approach” and shows means by gender ranging from 1 =“Strongly disagree” to 7 = “Strongly agree”. Sample size N = 1,291 (N = 468 men and N = 823 women). (PDF) [file pone.0225763.s006.pdf]

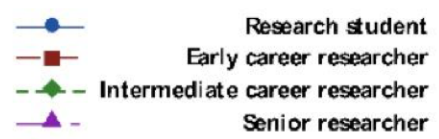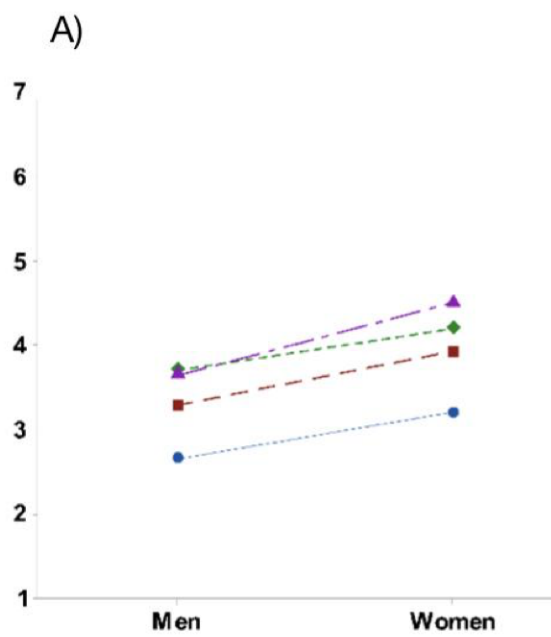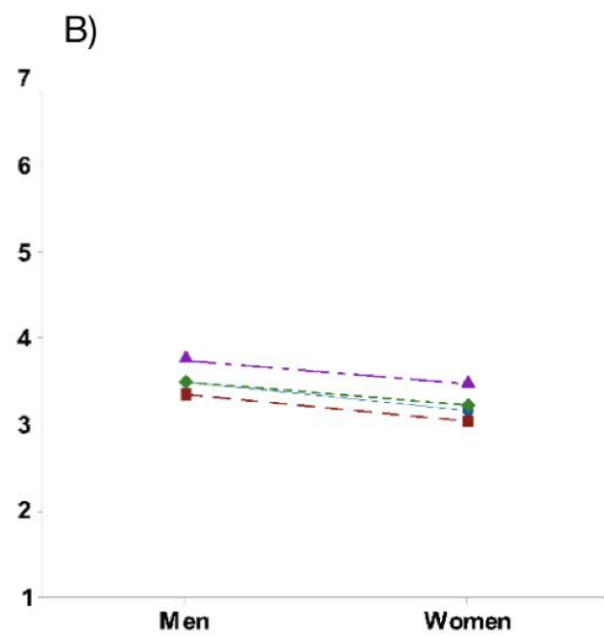

Supplement: S5 Fig — A) Appointments to editorships and B) Allocation of administrative tasks. Graph shows means by gender ranging from 1 =“Much easier for a woman” to 7 = “Much easier for a man”. Sample size N = 1,275 to 1,279 (N = from 462 to 463 men and N = from 813 to 816 women). (PDF) [file pone.0225763.s007.pdf]
