# Supplementary material for: Men and women differ in their perception of gender bias in research institutions
Source: PLoS One. 2019 Dec 5;14(12):e0225763. doi: 10.1371/journal.pone.0225763 (PMC6894819; doi:10.1371/journal.pone.0225763)
Supplement: S1 Table — (PDF) [file pone.0225763.s008.pdf]

Table S1. Description of the questions in the survey and variable names.

variable_name	Question	Values
Perceptions of gender equality in departments		
gender_eq_1	In general, men and women are treated equally in my department	1=strongly disagree to 7=strongly agree
gender_eq_2	My department is committed to promoting gender equality	
gender_eq_3	If I had concerns about gender equality in my department, I would know who to approach	
gender_eq_4	My department is (or would be) responsive to concerns about gender equality	
gender_eq_5	Women are perceived as good leaders in research by other women	
gender_eq_6	Women are perceived as good leaders in research by men	
Perceptions of gender equality in the allocation of tasks and resources		
gender_alloc_1	Allocation of desirable and demanded tasks/roles	1=Much easier for a woman to 7 Much easier for a man
gender_alloc_2	Receipt of mentoring	
gender_alloc_3	Attention from senior management	
gender_alloc_4	Access to informal circles of influence	
gender_alloc_5	Receiving positive feedback from management	
gender_alloc_6	Being recruited and selected for academic posts	
gender_alloc_7	Promotion to senior posts	
gender_alloc_8	Allocation of career development opportunities	
gender_alloc_9	Distribution of office/laboratory space or equipment	
gender_alloc_10	Invitations to conferences	
gender_alloc_11	Appointments to editorships	
gender_alloc_12	Recognition of intellectual contributions	
gender_alloc_13	Allocation of administrative tasks	
gender_alloc_14	Allocation of pastoral care roles	
gender_alloc_15	Allocation of teaching	
