# Supplementary material for: Men and women differ in their perception of gender bias in research institutions
Source: PLoS One. 2019 Dec 5;14(12):e0225763. doi: 10.1371/journal.pone.0225763 (PMC6894819; doi:10.1371/journal.pone.0225763)
Supplement: S2 Table — (PDF) [file pone.0225763.s009.pdf]

Table S2. Comparison between responses from female participants that did not complete the survey (excluded respondents) and participants included in the analysis (respondents that completed the survey).

Women	Respondents that completed the survey			Excluded respondents			Excluded respondents vs completed survey respondents				
	Mean	SD	N	Mean	SD	N	Difference in Means	SE of difference	t ratio	df	p value
gender eq 1	5.06	1.79	831	4.84	1.89	453	-0.22	0.11	2.08	1282	0.04
gender eq 2	4.32	1.93	832	4.42	2.02	451	0.10	0.11	0.88	1281	0.38
gender eq 3	3.90	2.14	829	3.83	2.15	446	-0.07	0.13	0.52	1273	0.60
gender eq 4	4.33	2.04	829	4.46	2.08	448	0.13	0.12	1.05	1275	0.30
gender eq 5	5.00	1.81	829	5.10	1.77	448	0.10	0.11	0.93	1275	0.35
gender eq 6	4.01	1.89	827	3.96	1.88	451	-0.05	0.11	0.46	1276	0.65
gender alloc 1	4.67	1.49	826	4.59	1.53	437	-0.08	0.09	0.87	1261	0.38
gender alloc 2	4.21	1.55	824	4.33	1.48	440	0.12	0.09	1.35	1262	0.18
gender alloc 3	4.65	1.55	820	4.78	1.42	441	0.13	0.09	1.49	1259	0.14
gender alloc 4	5.14	1.54	826	5.05	1.68	442	-0.09	0.09	0.97	1266	0.33
gender alloc 5	4.51	1.58	826	4.64	1.54	439	0.13	0.09	1.38	1263	0.17
gender alloc 6	4.95	1.34	827	4.81	1.57	442	-0.14	0.08	1.65	1267	0.10
gender alloc 7	5.28	1.59	824	5.27	1.57	441	-0.01	0.09	0.10	1263	0.92
gender alloc 8	4.96	1.48	827	4.93	1.57	440	-0.03	0.09	0.37	1265	0.71
gender alloc 9	4.27	1.18	825	4.21	1.32	441	-0.06	0.07	0.83	1264	0.41
gender alloc 10	4.60	1.58	825	4.58	1.54	440	-0.02	0.09	0.21	1263	0.83
gender alloc 11	4.00	2.08	819	4.00	2.08	432	0.00	0.12	0.01	1249	0.99
gender alloc 12	4.88	1.43	826	4.91	1.54	440	0.03	0.09	0.37	1264	0.71
gender alloc 13	3.25	1.43	822	3.30	1.53	432	0.06	0.09	0.65	1252	0.52
gender alloc 14	2.28	2.05	810	2.52	2.00	425	0.24	0.12	1.99	1233	0.05
gender alloc 15	3.71	1.31	826	3.67	1.50	437	-0.04	0.08	0.43	1261	0.67
