# Supplementary material for: Men and women differ in their perception of gender bias in research institutions
Source: PLoS One. 2019 Dec 5;14(12):e0225763. doi: 10.1371/journal.pone.0225763 (PMC6894819; doi:10.1371/journal.pone.0225763)
Supplement: S3 Table — (PDF) [file pone.0225763.s010.pdf]

**Table S3.** Comparison between responses from male participants that did not complete the survey (excluded respondents) and participants included in the analysis (respondents that completed the survey).

| Men | Respondents that completed the survey | | | Excluded respondents | | | Excluded respondents vs completed survey respondents | | | | |
|---|---|---|---|---|---|---|---|---|---|---|---|
| | Mean | Sd | N | Mean | Sd | N | Difference in Means | SE of difference | t ratio | df | p value |
| gender eq 1 | 6.04 | 1.43 | 472 | 6.10 | 1.29 | 229 | 0.06 | 0.11 | 0.54 | 699 | 0.59 |
| gender eq 2 | 4.97 | 1.93 | 471 | 5.14 | 2.04 | 229 | 0.17 | 0.16 | 1.07 | 698 | 0.28 |
| gender eq 3 | 4.51 | 2.18 | 472 | 4.34 | 2.29 | 228 | -0.18 | 0.18 | 0.99 | 698 | 0.32 |
| gender eq 4 | 5.07 | 1.91 | 471 | 4.86 | 2.18 | 227 | -0.21 | 0.16 | 1.28 | 696 | 0.20 |
| gender eq 5 | 5.06 | 2.26 | 468 | 5.01 | 2.21 | 224 | -0.05 | 0.18 | 0.27 | 690 | 0.79 |
| gender eq 6 | 5.39 | 1.77 | 470 | 5.29 | 1.82 | 225 | -0.10 | 0.14 | 0.68 | 693 | 0.50 |
| gender alloc 1 | 4.05 | 1.17 | 470 | 3.92 | 1.32 | 218 | -0.13 | 0.10 | 1.30 | 686 | 0.20 |
| gender alloc 2 | 3.85 | 1.05 | 468 | 3.73 | 1.34 | 217 | -0.13 | 0.09 | 1.34 | 683 | 0.18 |
| gender alloc 3 | 3.88 | 1.17 | 465 | 3.85 | 1.28 | 216 | -0.03 | 0.10 | 0.35 | 679 | 0.73 |
| gender alloc 4 | 4.13 | 1.51 | 467 | 4.08 | 1.70 | 219 | -0.06 | 0.13 | 0.43 | 684 | 0.67 |
| gender alloc 5 | 3.78 | 1.30 | 466 | 3.70 | 1.44 | 219 | -0.08 | 0.11 | 0.71 | 683 | 0.48 |
| gender alloc 6 | 4.03 | 1.29 | 469 | 4.00 | 1.27 | 219 | -0.03 | 0.10 | 0.28 | 686 | 0.78 |
| gender alloc 7 | 4.25 | 1.43 | 469 | 4.24 | 1.59 | 218 | -0.01 | 0.12 | 0.11 | 685 | 0.91 |
| gender alloc 8 | 4.06 | 1.34 | 467 | 4.00 | 1.40 | 216 | -0.05 | 0.11 | 0.46 | 681 | 0.65 |
| gender alloc 9 | 3.81 | 1.03 | 469 | 3.87 | 1.06 | 219 | 0.06 | 0.09 | 0.75 | 686 | 0.45 |
| gender alloc 10 | 3.91 | 1.17 | 467 | 3.62 | 1.55 | 219 | -0.29 | 0.11 | 2.72 | 684 | 0.01 |
| gender alloc 11 | 3.42 | 1.72 | 465 | 3.18 | 1.99 | 217 | -0.23 | 0.15 | 1.55 | 680 | 0.12 |
| gender alloc 12 | 4.06 | 1.14 | 467 | 4.06 | 1.45 | 219 | 0.01 | 0.10 | 0.06 | 684 | 0.95 |
| gender alloc 13 | 3.59 | 1.20 | 466 | 3.51 | 1.37 | 217 | -0.08 | 0.10 | 0.76 | 681 | 0.45 |
| gender alloc 14 | 2.44 | 2.00 | 458 | 2.68 | 1.98 | 213 | 0.24 | 0.17 | 1.43 | 669 | 0.15 |
| gender alloc 15 | 3.73 | 1.14 | 469 | 3.56 | 1.39 | 214 | -0.17 | 0.10 | 1.69 | 681 | 0.09 |
