# Supplementary material for: Men and women differ in their perception of gender bias in research institutions
Source: PLoS One. 2019 Dec 5;14(12):e0225763. doi: 10.1371/journal.pone.0225763 (PMC6894819; doi:10.1371/journal.pone.0225763)
Supplement: S4 Table — (PDF) [file pone.0225763.s011.pdf]

Table S4. List of the institutions represented in the sample analysed.

University/Centre name	Number of respondents
ABB	1
Agencia Aragonesa para la Investigación y el Desarrollo	1
AZTI	1
Basque Centre for Climate Change	3
Biodonostia Health Research Institute	1
Barcelona Supercomputer Center	1
Observatorio astronomico de Calar Alto	1
CCE	1
Centre de Recerca en Agrigenòmica	1
Centro Andaluz de Biología del Desarrollo	1
Centro Biologia Molecular Severo Ochoa	1
Centro de Biologia Molecular Severo Ochoa	6
Centro de Biología Molecular Severo Ochoa	5
Centro de Estudios de Fisica del Cosmos de Aragon	1
Centro de Investigación Príncipe Felipe	6
Centro de Investigación y Tecnología Agroalimentaria	1
Centro de Investigaciones Energéticas, Medioambientales y Tecnológicas	1
Centro de Regulación Genómica	1
Centro Nacional de Biotecnología	13
Centro Nacional de Métodos Numéricos en Ingeniería	1
Centro Universitario de la Defensa	1
Centro de Investigación Biomédica en Red	1
CIC bioGUNE	16
CIC BiomaGUNE	1
CIC nanoGUNE	2
Centro de *Investigaciones* Energéticas, Medioambientales y Tecnológicas	6
Clínica Universidad de Navarra	1
Centro Nacional de Investigaciones Cardiovasculares	7
Centro Nacional de Investigaciones Oncológicas	2
Colegio Agora	1
Centro de Investicación Agrigenómica UAB	3
Centro de Investigación Ecológica y Aplicaciones Forestales	1
Consejo Superior de Investigaciones Científicas	129
Centro Tecnológico de Telecomunicacions de Catalunya	2
Deusto	1
ESA	2
Estación Experimental del Zaidín	2
Fundación para la Investigación Médica Aplicada	1
Fundacio Scito	1
Fundación CTIC	1
Fundación Deusto	1
Fundacion para el Fomento de la Investigacion Sanitaria y Biomedica de la Comunidad Valenciana	1
Centro Pfizer-Universidad de Granada-Junta de Andalucía de Genómica e Investigación Oncológica	1
Henkel	1
Hospital	2
Hospital 12 de Octubre	3
Hospital Clinic de Barcelona	1
Hospital General Universitario Gregorio Marañon	1
Hospital Parc Taulí	2

Hospital Sant Joan de Déu	1
Hospital Universitario Central de Asturias	1
Hospital Universitario de La Princesa	1
Hospital Universitario de Las Palmas de Gran Canaria "Doctor Negrín"	1
Hospital Universitario Germans Trias i Pujol	1
Hospital Universitario Gregorio Marañón	1
Hospital Universitario Puerta de Hierro	1
Hospital Universitario Ramón y Cajal de Madrid	1
Hospital Virgen del Rocio	1
Institute of Biomedicine and Biotechnology of Cantabria	1
Institute for Bioengineering of Catalonia	1
Institute of Molecular Biology and Genetics IBGM	2
Instituto de Ciencias Matemáticas	1
Institut d'Investigació Biomédica de Bellvitge	2
Investigation en la Gestión de Organizaciones e Instalaciones Deportivas	1
Institute for Health Science Research Germans Trias i Pujol	1
Institutos Madrileños de Estudios Avanzados	1
Institutos Madrileños de Estudios Avanzados - Food	1
Institutos Madrileños de Estudios Avanzados - Nanociencia	1
Institutos Madrileños de Estudios Avanzados - Water	1
Instituto Madrileño de Investigación y Desarrollo Rural, Agrario y Alimentario	2
Instituto de Biotecnología de Leon	1
Incliva	1
Instituto Nacional de Investigación y Tecnología Agraria y Alimentaria	5
Insituto de Investigación Vall d'Hebron	3
Insituto de Investigaciones Biomedicas de Málaga	1
Institut Català de la Salut	1
Institut Català de Paleontologia Miquel Crusafont	1
Institut d'Estudis Espacials de Catalunya	1
Institut d'Investigació Germans Trias i Pujol	1
Institut de Recerca Biomèdica de Lleida	1
Institut de Rrecerca Sant Joan de Déu	1
Institute of Biomedical Research of Salamanca	1
Instituto Catalán de Arqueología Clásica	1
Instituto Catalán de investigación Química	1
Instituto Catalán de Investigación Química	16
Instituto Catalán de Neurociencia y Nanotechnology	14
Instituto de Astrofisica de Canarias	6
Instituto de Astrofísica de Canarias	2
Instituto de Biomedicina de Sevilla	3
Instituto de Ciencias Fotónicas	1
Instituto de Educación Física de Cataluña	1
Instituto de Educación Secundaria	1
Instituto de Fisica de Cantabria	1
Instituto de Física de Cantabria	1
Instituto de Investigación Biosanitaria de Granada	1
Instituto de Investigación contra la Leucemia Josep Carreras	12
Instituto de Investigación Sanitaria Galicia Sur	1
Instituto de Investigación Sanitaria Hospital 12 de Octubre	1
Instituto de Investigación Sanitaria La Fe	1
Instituto de Investigación Sanitaria Pere Virgili	1
Instituto de Investigación y Formación Agraria y Pesquera de Andalucía	1
Instituto de Investigaciones Biomédicas "Alberto Sols"	6
Instituto de Neurociencias de Alicante	1
Instituto de Neurociencias de Castilla y León	1

Instituto de Oncología de Asturias	1
Instituto de Salud Carlos III	5
Instituto Investigación Sanitaria Princesa	1
Instituto Maimónides de Investigación Biomédica de Córdoba	1
instituto Tecnológico Agrario de Castilla y León	1
Instituto Nacional de Técnica Aeroespacial	2
Intituto de investigaciones biomedicas de Barcelona (IIBB-CSIC)	1
Barcelona - Institute for Research in Biomedicine	6
Instituto Ramón y Cajal de Investigación Sanitaria (IRYCIS)	1
IsGlobal	1
Instituto de Investigación Sanitaria del Principado de Asturias	1
Laboratorios Syva	1
Leartiker S. Coop.	1
n-life Therapeutics	1
Neiker	1
Observatorio del Ebro	1
Polymat	1
Rey Juan Carlos University	1
SERGAS	2
Servicio Andaluz de Salud	1
SNiBA	1
Tecnalia	1
Universida de Vigo	1
Universidad Abierta de Cataluña	2
Universidad Antonio de Nebrija	11
Universidad Auntónoma de Madrid	1
Universidad Autónoma de Barcelona	133
Universidad Autonoma de Madrid	5
Universidad Autónoma de Madrid	10
Universidad Calos III	1
Universidad Carlos III de Madrid	52
Universidad Católica de Murcia	1
Universidad Complutende de Madrid	3
Universidad complutense de madrid	1
Universidad Complutense de madrid	1
Universidad Complutense de Madrid	29
Universidad Complutense Madrid	1
Universidad de A Coruña	4
Universidad de Alcala	1
Universidad de Alcalá	1
Universidad de Alicante	5
Universidad de Almeria	1
Universidad de Almería	1
Universidad de Barcelona	14
Universidad de BARCELONA	1
Universidad de Cádiz	1
Universidad de Cantabria	3
Universidad de Casilla - La Mancha	3
Universidad de Castilla la Mancha	1
Universidad de Castilla-La Mancha	43
Universidad de Córdoba	4
Universidad de Coruña	1
Universidad de Deusto	45
Universidad de Extremadura	4
Universidad de Girona	1

Universidad de Granada	9
Universidad de Jaén	1
Universidad de la Laguna	4
Universidad de La Rioja	5
Universidad de las Islas Baleares	5
Universidad de Las Palmas de Gran Canaria	3
Universidad de Leon	7
Universidad de León	31
Universidad de Lleida	14
Universidad de Malaga	2
Universidad de Málaga	1
Universidad de Mondragón	2
Universidad de Murcia	3
Universidad de Navarra	32
Universidad de Oviedo	2
Universidad de Rioja	1
Universidad de Salamanca	14
Universidad de Santiago de Compostela	9
Universidad de Sevilla	8
Universidad de Valencia	17
Universidad de Valladolid	62
Universidad de Vigo	45
Universidad de Zaragoza	25
Universidad del País Vasco	22
Universidad Europea	1
Universidad Internacional de Cataluña	1
Universidad Internacional de la Rioja	4
Universidad Miguel Hernandez	1
Universidad Nacional de Educación a Distancia	3
Universidad Pablo de Olavide	2
Universidad Politécnica de Cartagena	2
Universidad Politécnica de Cataluña	5
Universidad Politecnica de Madrid	3
Universidad Politécnica de Madrid	3
Universidad Politecnica de Valencia	2
Universidad Politécnica de Valencia	3
Universidad Pompeu Fabra	1
Universidad Publica de Navarra	1
Universidad Pública de Navarra	1
Universidad Ramón Llull	36
Universidad Rey Juan Carlos	39
Universidad Rovira i Virgili	2
Universidad Valladolid	1
Universidad Zaragoza	1
Universitat Autònoma Barcelona	2
Universitat Jaume I	1
Universitat Politecnica de Valencia	1
Universitat Pompeu Fabra	3
Vicomtech	1
**TOTAL**	**1221**
