# Supplementary material for: Men and women differ in their perception of gender bias in research institutions
Source: PLoS One. 2019 Dec 5;14(12):e0225763. doi: 10.1371/journal.pone.0225763 (PMC6894819; doi:10.1371/journal.pone.0225763)
Supplement: S5 Table — (PDF) [file pone.0225763.s012.pdf]

Table S5. Cronbach alpha coefficients for each item and whole category.

Variable	Adj. Total Mean	Adj. Total	Item-Adj. Total Corr	Squared Multiple	Cronbach's Alpha
**gender_eq_1**	22.805	7.035	0.5588	0.3967	0.7359
**gender_eq_2**	23.676	6.856	0.5707	0.3744	0.7304
**gender_eq_3**	24.106	6.878	0.4702	0.272	0.7579
**gender_eq_4**	23.629	6.817	0.5639	0.364	0.7318
**gender_eq_5**	23.194	7.166	0.3854	0.2567	0.7759
**gender_eq_6**	23.701	6.8	0.6052	0.4527	0.7216
Mean for category perceptions of gender equality in departments					0.7761

Variable	Adj. Total Mean	Adj. Total	Item-Adj. Total Corr	Squared Multiple	Cronbach's Alpha
**gender_alloc_1**	54.13	11.38	0.6004	0.4264	0.8476
**gender_alloc_2**	54.45	11.52	0.5164	0.3555	0.8522
**gender_alloc_3**	54.16	11.31	0.6423	0.4878	0.8452
**gender_alloc_4**	53.79	11.27	0.591	0.423	0.8476
**gender_alloc_5**	54.34	11.23	0.6504	0.4536	0.8442
**gender_alloc_6**	53.97	11.37	0.6193	0.528	0.8467
**gender_alloc_7**	53.67	11.17	0.656	0.603	0.8436
**gender_alloc_8**	53.95	11.21	0.6799	0.5573	0.8426
**gender_alloc_9**	54.46	11.65	0.5235	0.2874	0.8527
**gender_alloc_10**	54.23	11.4	0.5561	0.392	0.8498
**gender_alloc_12**	54	11.37	0.6289	0.4851	0.8462
**gender_alloc_13**	55.19	11.92	0.2122	0.1525	0.8671
**gender_alloc_14**	56.23	11.74	0.1851	0.1226	0.8786
**gender_alloc_15**	54.86	11.86	0.2865	0.1609	0.8631
Mean for category perceptions of gender equality in the allocation of tasks and resources					0.8614
