# Supplementary material for: Men and women differ in their perception of gender bias in research institutions
Source: PLoS One. 2019 Dec 5;14(12):e0225763. doi: 10.1371/journal.pone.0225763 (PMC6894819; doi:10.1371/journal.pone.0225763)
Supplement: S6 Table — “sd” = standard deviation. “N” = sample size. “df” = degrees of freedom. “95CI” = 95% Confidence intervals. (PDF) [file pone.0225763.s013.pdf]

Table S6. Descriptive and t-tests results for *perceptions of gender equality in departments*. “sd”=standard deviation. “N”=Sample size. “df”=degrees of freedom. “95CI”=95% Confidence intervals.

	Women			Men			Statistics				
**Question**	**mean**	**sd**	**N**	**mean**	**sd**	**N**	**t-value**	**df**	**95CI**	**P-Value**	**Cohen's d**
In general, men and women are treated equally in my department	5.06	1.79	825	6.05	1.41	468	-11.05	1160	0.8205 - 1.1748	2.20E-16	0.61
My department is committed to promoting gender equality	4.31	1.93	826	4.97	1.92	467	-5.95	973	0.444 - 0.881	3.72E-06	0.34
If I had concerns about gender equality in my department. I would know who to approach	3.9	2.15	823	4.52	2.17	468	-4.98	962	0.378 - 0.869	6.86E-07	0.28
My department is (or would be) responsive to concerns about gender equality	4.32	2.04	823	5.07	1.9	467	-6.61	1027	0.526 - 0.970	1.33E-10	0.38
Women are perceived as good leaders in research by other women	5.01	1.8	823	5.05	2.26	465	-0.28	799	0.205 - 0.274	7.79E-01	0.02
Women are perceived as good leaders in research by men	4.03	1.88	821	5.4	1.76	466	-13.11	1022	1.165 - 1.575	2.20E-16	0.75
**ALL ITEMS**	**4.44**	**1.93**	**812**	**5.18**	**1.90**	**461**	**7.42**	**2475**	**0.5204 – 0.9596**	**1.59E-13**	**0.39**
