# Supplementary material for: Men and women differ in their perception of gender bias in research institutions
Source: PLoS One. 2019 Dec 5;14(12):e0225763. doi: 10.1371/journal.pone.0225763 (PMC6894819; doi:10.1371/journal.pone.0225763)
Supplement: S7 Table — “sd” = standard deviation. “N” = sample size. “df” = degrees of freedom. “95CI” = 95% Confidence intervals. (PDF) [file pone.0225763.s014.pdf]

Table S7. Descriptive and t-tests results for *perceptions of gender equality in the allocation of tasks and resources*. "sd"=standard deviation. "N"=Sample size. "df"=degrees of freedom. "95CI"=95% Confidence intervals.

	Women			Men			Statistics				
**Question**	**mean**	**sd**	**N**	**mean**	**sd**	**N**	**t-value**	**df**	**95CI**	**P-Value**	**Cohen's d**
Allocation of desirable and demanded tasks/roles	4.67	1.49	820	4.05	1.16	467	8.33	1167	0.4759 - 0.7689	1.40E-14	0.46
Receipt of mentoring	4.22	1.54	818	3.87	1.04	465	4.84	1244	0.2080 - 0.4914	1.38E-05	0.27
Attention from senior management	4.67	1.53	814	3.87	1.17	462	10.37	1167	0.6427 - 0.9425	2.20E-16	0.58
Access to informal circles of influence	5.16	1.51	820	4.13	1.51	464	11.75	959	0.859 - 1.2039	2.20E-16	0.68
Receiving positive feedback from management	4.52	1.57	820	3.79	1.29	463	8.95	1118	0.568 - 0.8871	2.20E-16	0.51
Being recruited and selected for academic posts	4.96	1.32	821	4.04	1.27	466	12.29	993	0.7721 - 1.0654	2.20E-16	0.71
Promotion to senior posts	5.29	1.57	818	4.24	1.43	466	12.23	1045	0.8831 - 1.2205	2.20E-16	0.7
Allocation of career development opportunities	4.97	1.47	821	4.06	1.33	464	11.25	1040	0.7468 - 1.0624	2.20E-16	0.65
Distribution of office/laboratory space or equipment	4.27	1.17	819	3.81	1.02	466	7.32	1078	0.3350 - 0.5805	3.01E-12	0.42
**Average perception of gender equality in professional development**	**4.75**	**1.46**	793	**3.98**	**1.25**	454	8.24	6240	0.6098 - 0.9302	2.12E-16	0.57
Invitations to conferences	4.61	1.58	819	3.91	1.17	464	9.12	1191	0.5536 - 0.8571	2.20E-16	0.5
Appointments to editorships	4.01	2.07	813	3.42	1.71	462	5.53	1109	0.3840 - 0.807	1.78E-07	0.31

Recognition of intellectual contributions	4.88	1.43	820	4.07	1.13	464	11.15	1148	0.6658 - 0.9500	2.20E-16	0.63
Distribution of office/laboratory space or equipment	4.27	1.17	819	3.81	1.02	466	7.32	1078	0.3350 - 0.5805	3.01E-12	0.42
**Average perception of gender equality in markers of steem**	**4.44**	**1.69**	811	**3.80**	**1.34**	460	6.90	6240	0.4599 – 0.8201	5.57E-12	0.42
Allocation of administrative tasks	3.25	1.42	816	3.6	1.19	463	-4.67	1102	0.1969 – 0.5031	9.36E-06	0.27
Allocation of pastoral care roles	2.29	2.05	804	2.45	2	455	-1.32	961	0.3890 - 0.077	1.91E-01	0.08
Allocation of teaching	3.71	1.31	820	3.73	1.15	466	-0.21	1072	-0.2745	8.36E-01	0.02
**Average perception of gender equality in additional academic duties**	**3.09**	**1.59**	798	**3.26**	**1.45**	455	1.82	6240	-0.0076 – 0.3476	0.0684	0.11
**ALL ITEMS**	**4.33**	**1.54**	765	**3.80**	**1.30**	441	8.628	2475	0.3591- 0.7009	1.10E-17	0.37
