# Supplementary material for: Men and women differ in their perception of gender bias in research institutions
Source: PLoS One. 2019 Dec 5;14(12):e0225763. doi: 10.1371/journal.pone.0225763 (PMC6894819; doi:10.1371/journal.pone.0225763)
Supplement: S8 Table — (PDF) [file pone.0225763.s015.pdf]

Table S8. Research area variable names, sample size for each research area and gender distribution by research area.

Category Number	Total Sample size	Women/Men Sample size	Category name
1	378	252/126	Biological Sciences
2	196	137/59	Medical & Health Sciences
3	29	18/11	Business & Finance
4	83	52/31	Chemical Sciences
5	30	16/14	Earth Sciences
6	164	83/81	Engineering & computing
7	87	61/26	Humanities & Arts
8	28	19/9	Law
9	155	85/70	Maths & Physical Sciences
10	145	103/42	Social Sciences
