# Supplementary material for: Men and women differ in their perception of gender bias in research institutions
Source: PLoS One. 2019 Dec 5;14(12):e0225763. doi: 10.1371/journal.pone.0225763 (PMC6894819; doi:10.1371/journal.pone.0225763)
Supplement: S9 Table — “Df” = degrees of freedom. “Sum Sq” = Total sum of squares. “Mean Sq” = Mean Squares. (PDF) [file pone.0225763.s016.pdf]

Table S9. Interaction analysis of gender by research area in *perceptions of gender equality in departments. "*Df"=degrees of freedom. "Sum Sq"=Total sum of squares. "Mean Sq"=Mean Squares.

Item		Df	Sum Sq	Mean Sq	F value	P-value
Gender eq 1	gender	1	297,2	297,213	108,871	< 2.2e-16
	res_area	9	71,6	7,959	2,9155	0.002009
	gender:res_area	9	28,2	3,137	1,1492	0,324499
Gender eq 2	gender	1	130,8	130,835	35,4906	3.312e-09
	res_area	9	53,7	5,962	1,6173	0,1052
	gender:res_area	9	44,8	4,974	1,3493	0,2067
Gender eq 3	gender	1	116	115,958	24,9824	6.594e-07
	res_area	9	85,9	9,549	2,0573	0.03047
	gender:res_area	9	16,8	1,87	0,4028	0,93404
Gender eq 4	gender	1	166,6	166,584	41,8242	1.421e-10
	res_area	9	22,7	2,522	0,6331	0,7694
	gender:res_area	9	34,1	3,785	0,9504	0,48
Gender eq 5	gender	1	0,3	0,348	0,0901	0,76415
	res_area	9	83,8	9,3162	2,4106	0.01034
	gender:res_area	9	46,8	5,198	1,345	0,20878
Gender eq 6	gender	1	558,1	558,11	166,7301	< 2e-16
	res_area	9	46,8	5,2	1,5541	0,12418
	gender:res_area	9	51	5,67	1,6938	0.08571
