# Supplementary material for: Men and women differ in their perception of gender bias in research institutions
Source: PLoS One. 2019 Dec 5;14(12):e0225763. doi: 10.1371/journal.pone.0225763 (PMC6894819; doi:10.1371/journal.pone.0225763)
Supplement: S10 Table — “Df” = degrees of freedom. “Sum Sq” = Total sum of squares. “Mean Sq” = Mean Squares. (PDF) [file pone.0225763.s017.pdf]

Table S10. Interaction analysis of gender by research area in the *perceptions of gender equality in the allocation of tasks and resources. "*Df"=degrees of freedom. "Sum Sq"=Total sum of squares. "Mean Sq"=Mean Squares.

Item		Df	Sum Sq	Mean Sq	F value	P-value
Gender alloc 1	gender	1	115,26	115,264	60,846	1.282e-14
	res_area	9	17,98	1,998	1,0546	0,394
	gender:res_area	9	24,26	2,696	1,423	0,1729
Gender alloc 2	gender	1	36,26	36,258	19,0526	1.375e-05
	res_area	9	12,72	1,413	0,7427	0,6699
	gender:res_area	9	22,15	2,461	1,293	0,2358
Gender alloc 3	gender	1	185,16	185,158	93,2697	<2e-16
	res_area	9	18,74	2,083	1,0491	0,3983
	gender:res_area	9	23,35	2,595	1,307	0,2283
Gender alloc 4	gender	1	315,39	315,387	138,2071	<2e-16
	res_area	9	24,64	2,737	1,1995	0,2911
	gender:res_area	9	13,92	1,547	0,6778	0,7297
Gender alloc 5	gender	1	156,62	156,622	71,4476	<2e-16
	res_area	9	11,51	1,279	0,5836	0,8114
	gender:res_area	9	13,88	1,542	0,7033	0,7063
Gender alloc 6	gender	1	250,92	250,922	147,9367	<2e-16
	res_area	9	22,63	2,515	1,4827	0,149
	gender:res_area	9	3,17	0,352	0,2074	0,9934
Gender alloc 7	gender	1	328,45	328,45	142,3545	< 2e-16
	res_area	9	43,79	4,87	2,1087	0.02615
	gender:res_area	9	6,09	0,68	0,2933	0,97676
Gender alloc 8	gender	1	242,6	242,596	120,8565	< 2.2e-16
	res_area	9	50,31	5,59	2,7851	0.003097
	gender:res_area	9	5,74	0,638	0,3178	0,969425
Gender alloc 9	gender	1	62,24	62,235	50,0823	2.436e-12
	res_area	9	32,16	3,573	2,8752	0.002298
	gender:res_area	9	4,46	0,495	0,3985	0,936174
Gender alloc 10	gender	1	147,35	147,353	71,0624	< 2e-16
	res_area	9	33,97	3,774	1,8201	0.06049
	gender:res_area	9	13,7	1,522	0,734	0,67792
Gender alloc 11	gender	1	104,6	104,567	27,8382	1.552e-07
	res_area	9	78	8,662	2,3061	0.01434
	gender:res_area	9	37,2	4,13	1,0994	0,36003
Gender alloc 12	gender	1	193,39	193,394	109,6944	<2e-16
	res_area	9	25,64	2,848	1,6156	0,1057
	gender:res_area	9	10,25	1,139	0,6462	0,758
Gender alloc 13	gender	1	35,58	35,578	19,7692	9.508e-06
	res_area	9	18	2	1,1113	0,3513

	gender:res_area	9	11,02	1,224	0,6804	0,7273
	gender	1	7,1	7,1032	1,7341	0,188125
Gender alloc 14	res_area	9	110,4	12,2694	2,9954	0.001541
	gender:res_area	9	28,5	3,1619	0,7719	0,642585
	gender	1	0,07	0,06667	0,0427	0,8363
Gender alloc 15	res_area	9	22,42	2,49122	1,5966	0,1111
	gender:res_area	9	7,3	0,81133	0,52	0,8609
