# Supplementary material for: Men and women differ in their perception of gender bias in research institutions
Source: PLoS One. 2019 Dec 5;14(12):e0225763. doi: 10.1371/journal.pone.0225763 (PMC6894819; doi:10.1371/journal.pone.0225763)
Supplement: S11 Table — (PDF) [file pone.0225763.s018.pdf]

Table S11. Position variable names, sample size for each position and gender distribution by position.

Category Number	Total Sample size	Women/Men Sample size	Category name
0	51	40/11	Other
1	560	311/245	Research student
2	214	141/71	Early career researcher
3	213	153/59	Intermediate career researcher
4	268	181/83	Senior researcher
