# Supplementary material for: Men and women differ in their perception of gender bias in research institutions
Source: PLoS One. 2019 Dec 5;14(12):e0225763. doi: 10.1371/journal.pone.0225763 (PMC6894819; doi:10.1371/journal.pone.0225763)
Supplement: S12 Table — “Df” = degrees of freedom. “Sum Sq” = total sum of squares. “Mean Sq” = Mean Squares. (PDF) [file pone.0225763.s019.pdf]

Table S12. Interaction analysis of gender by position in the *perceptions of gender equality in departments. "*Df"=degrees of freedom. "Sum Sq"=Total sum of squares. "Mean Sq"=Mean Squares.

Item		Df	Sum Sq	Mean Sq	F value	P-value
Gender eq 1	gender	1	211.865	211.856	77.07	0.000
	position	3	19.55	6.511	2.37	0.069
	age	1	1	1.200	0.43	0.5100
	gender:age	1	12	11.700	4.23	0.0399
	gender:position	3	11.44	3.813	1.39	0.245
Gender eq 2	gender	1	64.71	64.712	17.53	0.000
	position	3	38.72	12.905	3.50	0.015
	age	1	12	12.310	3.33	0.0684
	gender:age	1	15	14.880	4.02	0.0452
	gender:position	3	17.15	5.717	1.55	0.200
Gender eq 3	gender	1	90.01	90.014	19.64	0.000
	position	3	77.30	25.767	5.62	0.001
	age	1	94	94.280	20.53	<0.0001
	gender:age	1	21	20.890	4.55	0.0331
	gender:position	3	14.37	4.791	1.05	0.371
Gender eq 4	gender	1	112.11	112.111	28.36	0.000
	position	3	44.74	14.913	3.77	0.010
	age	1	35	35.330	8.92	0.0029
	gender:age	1	0	0.080	0.02	0.8856
	gender:position	3	10.17	3.391	0.86	0.462
Gender eq 5	gender	1	0.49	0.4918	0.13	0.723
	position	3	12.61	4.2043	1.07	0.359
	age	1	4	4.245	1.09	0.2980
	gender:age	1	5	5.205	1.33	0.2490
	gender:position	3	18.52	6.1735	1.58	0.193
Gender eq 6	gender	1	400.91	400.910	119.21	0.000
	position	3	28.88	9.626	2.86	0.036
	age	1	28	28.400	8.43	0.0038
	gender:age	1	7	6.900	2.06	0.5100
	gender:position	3	15.28	5.092	1.51	0.209
