# Supplementary material for: Men and women differ in their perception of gender bias in research institutions
Source: PLoS One. 2019 Dec 5;14(12):e0225763. doi: 10.1371/journal.pone.0225763 (PMC6894819; doi:10.1371/journal.pone.0225763)
Supplement: S13 Table — “Df” = degrees of freedom. “Sum Sq” = total sum of squares. “Mean Sq” = mean Squares. (PDF) [file pone.0225763.s020.pdf]

Table S13. Interaction of gender by position in the *perceptions of gender equality in allocation of tasks and resources.* *"*Df"=degrees of freedom. "Sum Sq"=Total sum of squares. "Mean Sq"=Mean Squares.

Item		Df	Sum Sq	Mean Sq	F value	P-value
Gender alloc 1	gender	1	79.74	79.736	42.58	0.000
	position	3	22.99	7.663	4.09	0.007
	age	1	22.8	22.770	12.20	0.0005
	gender:age	1	10.9	10.890	5.84	0.0158
	gender:position	3	10.59	3.532	1.89	0.130
Gender alloc 2	gender	1	21.72	21.717	11.47	0.001
	position	3	10.66	3.553	1.88	0.132
	age	1	12.6	12.560	6.67	0.0099
	gender:age	1	10.1	10.110	5.37	0.0207
	gender:position	3	9.20	3.067	1.62	0.183
Gender alloc 3	gender	1	130.32	130.316	66.12	0.000
	position	3	13.50	4.500	2.28	0.077
	age	1	6.9	6.940	3.53	0.0604
	gender:age	1	15.6	15.580	7.92	0.0050
	gender:position	3	11.21	3.736	1.90	0.128
Gender alloc 4	gender	1	235.50	235.499	104.72	0.000
	position	3	29.83	9.943	4.42	0.004
	age	1	21.6	21.600	9.70	0.0019
	gender:age	1	20.8	20.800	9.35	0.0023
	gender:position	3	15.15	5.051	2.25	0.081
Gender alloc 5	gender	1	100.30	100.303	46.53	0.000
	position	3	6.48	2.160	1.00	0.391
	age	1	6.5	6.550	3.05	0.0810
	gender:age	1	30.8	30.830	14.36	0.0002
	gender:position	3	22.06	7.352	3.41	0.017
Gender alloc 6	gender	1	216.83	216.833	127.91	0.000
	position	3	1.64	0.546	0.32	0.809
	age	1	1.8	1.760	1.05	0.3070
	gender:age	1	1.4	1.390	0.83	0.3640
	gender:position	3	6.32	2.108	1.24	0.293
Gender alloc 7	gender	1	274.21	274.206	118.03	0.000
	position	3	3.62	1.206	0.52	0.669
	age	1	0.8	0.800	0.36	0.5500
	gender:age	1	3.1	3.100	1.34	0.2480
	gender:position	3	2.64	0.878	0.38	0.769
Gender alloc 8	gender	1	189.22	189.215	93.24	0.000

	position	3	9.73	3.243	1.60	0.188
	age	1	3.5	3.530	1.75	0.1860
	gender:age	1	2.4	2.360	1.17	0.2790
	gender:position	3	1.72	0.573	0.28	0.838
Gender alloc 9	gender	1	44.51	44.506	35.85	0.000
	position	3	17.01	5.670	4.57	0.003
	age	1	13.8	13.800	11.21	0.0008
	gender:age	1	8.7	8.710	7.07	0.0079
	gender:position	3	3.93	1.311	1.06	0.367
Gender alloc 10	gender	1	114.40	114.401	55.69	0.000
	position	3	23.63	7.877	3.83	0.009
	age	1	25.3	25.290	12.40	0.0004
	gender:age	1	12.8	12.800	6.27	0.0124
	gender:position	3	15.10	5.035	2.45	0.062
Gender alloc 11	gender	1	99.26	99.2601	27.30	0.000
	position	3	195.96	65.3186	17.97	0.000
	age	1	132	132.100	36.05	<0.0001
	gender:age	1	6	5.640	1.54	0.2150
	gender:position	3	1.77	0.5915	0.16	0.921
Gender alloc 12	gender	1	158.83	158.835	91.20	0.000
	position	3	14.90	4.967	2.85	0.036
	age	1	7.9	7.930	4.55	0.0332
	gender:age	1	9.6	9.630	5.52	0.0189
	gender:position	3	6.41	2.136	1.23	0.299
Gender alloc 13	gender	1	21.37	21.3735	12.04	0.001
	position	3	33.66	11.2185	6.32	0.000
	age	1	36.3	36.260	20.42	<0.0001
	gender:age	1	1.8	1.780	1.00	0.3170
	gender:position	3	0.97	0.3224	0.18	0.909
Gender alloc 14	gender	1	1.67	1.672	0.40	0.525
	position	3	38.55	12.848	3.11	0.026
	age	1	23	23.151	5.60	0.0181
	gender:age	1	3	3.097	0.75	0.3870
	gender:position	3	10.84	3.612	0.87	0.454
Gender alloc 15	gender	1	0.01	0.1217	0.01	0.929
	position	3	10.92	3.64107	2.35	0.071
	age	1	5	4.990	3.20	0.0739
	gender:age	1	1.1	1.126	0.72	0.3957
	gender:position	3	2.32	0.77397	0.50	0.683
