# Supplementary material for: Men and women differ in their perception of gender bias in research institutions
Source: PLoS One. 2019 Dec 5;14(12):e0225763. doi: 10.1371/journal.pone.0225763 (PMC6894819; doi:10.1371/journal.pone.0225763)
Supplement: S14 Table — (PDF) [file pone.0225763.s021.pdf]

Table S14. Type of institution variable names, sample size for each type of institution and gender distribution.

**Category Number**	**Total Sample size**	**Women/Men Sample size**	**Category name**
1	691	432/259	Public university
2	136	89/47	Private university
3	342	219/123	Research centre
