# Supplementary material for: Men and women differ in their perception of gender bias in research institutions
Source: PLoS One. 2019 Dec 5;14(12):e0225763. doi: 10.1371/journal.pone.0225763 (PMC6894819; doi:10.1371/journal.pone.0225763)
Supplement: S15 Table — “Df” = degrees of freedom. “Sum Sq” = total sum of squares. “Mean Sq” = mean Squares. (PDF) [file pone.0225763.s022.pdf]

Table S15. Interaction analysis of gender by type of institution in the *perceptions of gender equality in departments. "*Df"=degrees of freedom. "Sum Sq"=Total sum of squares. "Mean Sq"=Mean Squares.

Item		Df	Sum Sq	Mean Sq	F value	P-value
Gender eq 1	gender	1	65.52	654.163	23.66	0.000
	institution type	1	0.73	0.7280	0.26	0.608
	gender:institution type	1	0.54	0.5360	0.19	0.660
Gender eq 2	gender	1	12.19	121.865	3.32	0.069
	institution type	1	0.01	0.0147	0.00	0.950
	gender:institution type	1	4.07	40.692	1.11	0.293
Gender eq 3	gender	1	27.51	275.129	6.01	0.014
	institution type	1	6.08	60.791	1.33	0.250
	gender:institution type	1	0.59	0.5859	0.13	0.721
Gender eq 4	gender	1	30.29	302.892	7.83	0.005
	institution type	1	0.27	0.2687	0.07	0.792
	gender:institution type	1	0.08	0.0772	0.02	0.888
Gender eq 5	gender	1	0.67	0.66899	0.17	0.678
	institution type	1	0.01	0.01223	0.00	0.955
	gender:institution type	1	0.65	0.64942	0.17	0.683
Gender eq 6	gender	1	97.60	97.598	28.93	0.000
	institution type	1	0.03	0.035	0.01	0.919
	gender:institution type	1	0.08	0.076	0.02	0.881
