# Supplementary material for: Men and women differ in their perception of gender bias in research institutions
Source: PLoS One. 2019 Dec 5;14(12):e0225763. doi: 10.1371/journal.pone.0225763 (PMC6894819; doi:10.1371/journal.pone.0225763)
Supplement: S16 Table — “Df” = degrees of freedom. “Sum Sq” = Total sum of squares. “Mean Sq” = Mean Squares. (PDF) [file pone.0225763.s023.pdf]

**Table S16.** Interaction analysis of gender by type of institution in the *perceptions of gender equality in allocation of tasks and resources. "*Df"=degrees of freedom. "Sum Sq"=Total sum of squares. "Mean Sq"=Mean Squares.

| Item | | Df | Sum Sq | Mean Sq | F value | P-value |
|---|---|---|---|---|---|---|
| Gender alloc 1 | gender | 1 | 30.50 | 30.500 | 15.87 | 0.000 |
| | institution type | 1 | 1.61 | 1.612 | 0.84 | 0.360 |
| | gender:institution type | 1 | 1.58 | 1.580 | 0.82 | 0.365 |
| Gender alloc 2 | gender | 1 | 18.70 | 18.703 | 9.71 | 0.002 |
| | institution type | 1 | 3.90 | 3.895 | 2.02 | 0.155 |
| | gender:institution type | 1 | 3.87 | 3.868 | 2.01 | 0.157 |
| Gender alloc 3 | gender | 1 | 28.36 | 28.360 | 14.25 | 0.000 |
| | institution type | 1 | 1.12 | 1.117 | 0.56 | 0.454 |
| | gender:institution type | 1 | 0.57 | 0.567 | 0.29 | 0.593 |
| Gender alloc 4 | gender | 1 | 72.65 | 72.652 | 31.97 | 0.000 |
| | institution type | 1 | 0.75 | 0.746 | 0.33 | 0.567 |
| | gender:institution type | 1 | 0.90 | 0.896 | 0.39 | 0.530 |
| Gender alloc 5 | gender | 1 | 39.12 | 39.115 | 18.30 | 0.000 |
| | institution type | 1 | 0.16 | 0.160 | 0.08 | 0.784 |
| | gender:institution type | 1 | 0.61 | 0.612 | 0.29 | 0.593 |
| Gender alloc 6 | gender | 1 | 43.82 | 42.815 | 25.78 | 0.000 |
| | institution type | 1 | 0.57 | 0.570 | 0.34 | 0.562 |
| | gender:institution type | 1 | 0.17 | 0.171 | 0.10 | 0.751 |
| Gender alloc 7 | gender | 1 | 44.98 | 44.979 | 19.43 | 0.000 |
| | institution type | 1 | 4.73 | 4.731 | 2.04 | 0.153 |
| | gender:institution type | 1 | 2.33 | 2.325 | 1.00 | 0.316 |
| Gender alloc 8 | gender | 1 | 44.98 | 44.979 | 19.43 | 0.000 |
| | institution type | 1 | 4.73 | 4.731 | 2.04 | 0.153 |
| | gender:institution type | 1 | 2.33 | 2.325 | 1.00 | 0.316 |
| Gender alloc 9 | gender | 1 | 41.44 | 41.443 | 20.57 | 0.000 |
| | institution type | 1 | 0.01 | 0.0135 | 0.01 | 0.935 |
| | gender:institution type | 1 | 0.15 | 0.148 | 0.07 | 0.786 |
| Gender alloc 10 | gender | 1 | 58.15 | 58.154 | 28.59 | 0.000 |
| | institution type | 1 | 3.88 | 3.882 | 1.91 | 0.167 |
| | gender:institution type | 1 | 4.76 | 4.764 | 2.34 | 0.126 |
| Gender alloc 11 | gender | 1 | 32.63 | 32.633 | 8.62 | 0.003 |
| | institution type | 1 | 5.85 | 5.851 | 1.54 | 0.214 |
| | gender:institution type | 1 | 1.32 | 1.316 | 0.35 | 0.556 |
| Gender alloc 12 | gender | 1 | 46.22 | 46.215 | 26.78 | 0.000 |
| | institution type | 1 | 0.13 | 0.127 | 0.07 | 0.786 |
| | gender:institution type | 1 | 0.13 | 0.129 | 0.08 | 0.784 |
| Gender alloc 13 | gender | 1 | 1.20 | 1.202 | 0.68 | 0.410 |
| | institution type | 1 | 6.42 | 6.417 | 3.63 | 0.057 |
| | gender:institution type | 1 | 2.84 | 2.839 | 1.61 | 0.205 |
| Gender alloc 14 | gender | 1 | 0.05 | 0.052 | 0.01 | 0.911 |
| | institution type | 1 | 1.35 | 1.351 | 0.32 | 0.571 |
| | gender:institution type | 1 | 1.11 | 1.113 | 0.26 | 0.607 |
| Gender alloc 15 | gender | 1 | 0.62 | 0.623 | 0.43 | 0.514 |
| | institution type | 1 | 7.99 | 7.992 | 5.47 | 0.020 |
| | gender:institution type | 1 | 0.38 | 0.383 | 0.26 | 0.609 |
