# Supplementary material for: Men and women differ in their perception of gender bias in research institutions
Source: PLoS One. 2019 Dec 5;14(12):e0225763. doi: 10.1371/journal.pone.0225763 (PMC6894819; doi:10.1371/journal.pone.0225763)
Supplement: S17 Table — “N” = Sample size, “M” = mean, “SD” = Standard deviation, “df” = degrees of freedom. Note: The questions “women are perceived as good leaders by women/men” from the Spain based questionnaire are not reported in this analysis, as no equivalent questions were available in ASSET 2016. Significance declared at Bonferroni corrected threshold p = 0.001. (PDF) [file pone.0225763.s024.pdf]

Table S17. Comparison of responses between Spain vs the United Kingdom-based researchers. "N"=Sample size, "M"=mean, "SD"=Standard deviation, "df"=degrees of freedom. Note: The questions "women are perceived as good leaders by women/men" from the Spain based questionnaire are not reported in this analysis, as no equivalent questions were available in ASSET 2016. Significance declared at Bonferroni corrected threshold p=0.001.

Item code	Gender	N	M	SD	N	M	SD	Statistics			
			UK			Spain		t-value	df	P-Value	Adj.P-value
gender alloc 1	Women	2351	4.56	0.906	820	4.67	1.49	2.494	3169	0.013	0.000309126
gender alloc 1	Men	2482	3.99	0.643	467	4.05	1.16	1.588	2947	0.112	0.002739283
gender alloc 10	Women	2334	4.53	0.916	819	4.61	1.58	1.748	3151	0.080	0.001963275
gender alloc 10	Men	2476	3.92	0.713	464	3.91	1.17	0.2463	2938	0.805	0.0196449
gender alloc 11	Women	2346	4.16	0.644	813	4.01	2.07	3.104	3157	0.002	4.70144E-05
gender alloc 11	Men	2467	3.89	0.553	462	3.42	1.71	10.94	2927	<0.001	<2.43902E-17
gender alloc 12	Women	2324	4.18	0.707	820	4.88	1.43	18.14	3142	<0.001	<2.43902E-17
gender alloc 12	Men	2462	3.97	0.409	464	4.07	1.13	3.374	2924	0.001	1.83129E-05
gender alloc 13	Women	2298	4.19	0.561	816	3.25	1.42	26.45	3112	<0.001	<2.43902E-17
gender alloc 13	Men	2458	4	0.381	463	3.60	1.19	13.42	2919	<0.001	<2.43902E-17
gender alloc 14	Women	2330	4.33	0.744	804	2.29	2.05	40.87	3132	<0.001	<2.43902E-17
gender alloc 14	Men	2462	4	0.523	455	2.45	2.00	32.87	2915	<0.001	<2.43902E-17
gender alloc 15	Women	2289	4.23	0.607	820	3.71	1.31	15.02	3107	<0.001	<2.43902E-17
gender alloc 15	Men	2433	4.05	0.4	466	3.73	1.15	10.75	2897	<0.001	<2.43902E-17
gender alloc 2	Women	2349	4.21	0.839	814	4.67	1.53	10.66	3161	<0.001	<2.43902E-17
gender alloc 2	Men	2475	3.87	0.616	465	3.87	1.04	0	2938	<0.001	<2.43902E-17
gender alloc 3	Women	2345	4.07	1.127	820	4.67	1.51	11.86	3157	<0.001	<2.43902E-17
gender alloc 3	Men	2472	3.92	0.612	462	3.87	1.17	1.354	2932	0.176	0.004289638
gender alloc 4	Women	2344	3.92	0.992	820	4.52	1.57	12.65	3162	<0.001	<2.43902E-17
gender alloc 4	Men	2472	3.88	0.586	464	4.13	1.51	6.134	2934	<0.001	2.3762E-11
gender alloc 5	Women	2344	4.55	0.93	821	4.96	1.32	9.673	3163	<0.001	<2.43902E-17
gender alloc 5	Men	2477	3.92	0.721	463	3.79	1.29	3.069	2938	0.002	5.28044E-05

gender alloc 6	Women	2343	4.78	1.036	818	5.29	1.57	10.49	3159	<0.001	<2.43902E-17
gender alloc 6	Men	2471	4.08	0.783	466	4.04	1.27	0.9017	2935	0.367	0.008958039
gender alloc 7	Women	2342	4.32	0.769	821	4.97	1.47	16.04	3161	<0.001	<2.43902E-17
gender alloc 7	Men	2467	3.94	0.554	466	4.24	1.43	7.781	2931	<0.001	2.43902E-16
gender alloc 8	Men	2476	3.97	0.727	464	4.06	1.33	2.87	3164	0.004	0.000100911
gender alloc 8	Women	2347	4.38	0.852	819	4.27	1.17	2.091	2938	0.037	0.000893229
gender alloc 9	Women	2356	4.24	0.664	818	4.22	1.54	0.5088	3172	0.611	0.014900666
gender alloc 9	Men	2483	3.99	0.409	466	3.81	1.02	6.456	2947	<0.001	3.06476E-12
**ALL ITEMS ALLOCATION**	Women	**2324**	**4.31**	**0.82**	**765**	**4.33**	**1.54**	**0.3545**	**12070**	**0.723**	
	Men	**2433**	**3.96**	**0.58**	**441**	**3.80**	**1.30**	**2.2839**	**12070**	**0.022**	
gender eq 1	Women	2371	4.81	1.78	825	5.06	1.79	3.47	3194	0.001	1.28814E-05
gender eq 1	Men	2491	5.73	1.522	468	6.05	1.41	4.221	2957	<0.001	6.1177E-07
gender eq 2	Women	2363	5.14	1.538	826	4.31	1.93	12.46	3187	<0.001	<2.43902E-17
gender eq 2	Men	2491	5.82	1.361	467	4.97	1.92	11.52	2956	<0.001	<2.43902E-17
gender eq 3	Women	2367	4.81	1.906	823	3.90	2.15	11.4	3188	<0.001	<2.43902E-17
gender eq 3	Men	2487	5.51	1.692	468	4.52	2.17	11.06	2953	<0.001	<2.43902E-17
gender eq 4	Women	2364	5	1.624	823	4.32	2.04	9.651	3185	<0.001	<2.43902E-17
gender eq 4	Men	2479	5.73	1.372	467	5.07	1.90	8.911	2944	<0.001	<2.43902E-17
**ALL ITEMS GENDER EQUALITY**	Women	**2363**	**4.94**	**1.71**	**812**	**4.40**	**1.98**	**9.807**	**12070**	**1.27E-22**	
	Men	**2479**	**5.70**	**1.49**	**461**	**5.15**	**1.85**	**8.011**	**12070**	**1.23E-15**	
